# Impact of Life History on Fear Memory and Extinction

**DOI:** 10.3389/fnbeh.2016.00185

**Published:** 2016-10-04

**Authors:** Jasmin Remmes, Carina Bodden, S. Helene Richter, Jörg Lesting, Norbert Sachser, Hans-Christian Pape, Thomas Seidenbecher

**Affiliations:** ^1^Institute of Physiology I, Westfälische Wilhelms-UniversityMünster, Germany; ^2^Otto Creutzfeldt Center for Cognitive and Behavioral Neuroscience, Westfälische Wilhelms-UniversityMünster, Germany; ^3^Department of Behavioural Biology, Westfälische Wilhelms-UniversityMünster, Germany

**Keywords:** anxiety, conditioning, behavior, fear learning, freezing, allostatic load, mismatch

## Abstract

Behavioral profiles are strongly shaped by an individual's whole life experience. The accumulation of negative experiences over lifetime is thought to promote anxiety-like behavior in adulthood (“allostatic load hypothesis”). In contrast, the “mismatch hypothesis” of psychiatric disease suggests that high levels of anxiety-like behavior are the result of a discrepancy between early and late environment. The aim of the present study was to investigate how different life histories shape the expression of anxiety-like behavior and modulate fear memory. In addition, we aimed to clarify which of the two hypotheses can better explain the modulation of anxiety and fear. For this purpose, male mice grew up under either adverse or beneficial conditions during early phase of life. In adulthood they were further subdivided in groups that either matched or mismatched the condition experienced before, resulting in four different life histories. The main results were: (i) Early life benefit followed by late life adversity caused decreased levels of anxiety-like behavior. (ii) Accumulation of adversity throughout life history led to impaired fear extinction learning. Late life adversity as compared to late life benefit mainly affected extinction training, while early life adversity as compared to early life benefit interfered with extinction recall. Concerning anxiety-like behavior, the results do neither support the allostatic load nor the mismatch hypothesis, but rather indicate an anxiolytic effect of a mismatched early beneficial and later adverse life history. In contrast, fear memory was strongly affected by the accumulation of adverse experiences over the lifetime, therefore supporting allostatic load hypothesis. In summary, this study highlights that anxiety-like behavior and fear memory are differently affected by specific combinations of adverse or beneficial events experienced throughout life.

## Introduction

Development of fear and anxiety in an adult individual are thought to be critically shaped by its environmental conditions. It is conventionally accepted that cumulative stress throughout lifetime poses one of the predominant environmental risks for the development of several psychiatric disorders (anxiety disorders, phobias, posttraumatic stress disorders (PTSD), depression, etc.). So far, different hypotheses exist which address the correlation between early life socio-environmental stressors and the individuals' vulnerability to psychiatric diseases (de Kloet et al., 2005; McEwen, 2006; Taylor, 2010). One acknowledged hypothesis suggests that an accumulation of negative events results in an increase of the subjects' allostatic load over lifetime. This effect is described as “cumulative stress” or “allostatic load” hypothesis and is particularly detrimental since it is considered to augment the susceptibility to express anxiety-related behaviors (McEwen, 2003). On the contrary, overall positive experiences are thought to facilitate learning and resilience, leaving the individual less vulnerable to develop an anxious phenotype (Doulames et al., 2014).

Beyond anxiety-related behaviors, fear responses are equally observed across many species. Fear and anxiety are crucial to evoke behavioral responses, such as avoidance or caution upon threatening stimuli to protect the individual from potential harm. In general, fear responses are related to the presence of a particular cue or context, whereas anxiety is a more diffuse state that can occur irrespective of these triggering elements. Both emotional states result in specific and measurable reactions, such as flight, freezing, risk-assessment, or reduced locomotor activity. Therefore, a broad variety of standard procedures exists to differentiate between fear and anxiety on the behavioral level (e.g., open field-, elevated plus-maze test, Pavlovian fear conditioning; for review see Tovote et al., 2015). Especially Pavlovian fear conditioning has become one of the standard behavioral paradigms to study explicitly emotional learning and fear memory processes (LeDoux, 1993; for review see Pape and Pare, 2010). In such a conditioned fear paradigm, a neutral stimulus (e.g., tone or light) is paired with an aversive stimulus (e.g., electric footshock) to trigger fear responses on demand, which provides a measure for fear learning *per se* and also facilitates the subject to predict possible environmental threats (LeDoux, 2000). These fear responses typically decline if the subject is exposed to repetitive presentations of non-reinforced cues where it learns that the conditioned stimulus (CS) no longer predicts danger, a physiological response, called fear extinction (Maren and Quirk, 2004). Fear and anxiety are part of an organisms' defensive mechanism and critical for survival. Yet, pathological expression of fear and anxiety brain states are shown to modulate neuronal activities and lead to behavioral changes (Calhoon and Tye, 2015).

It is known that neuronal networks are shaped by the social environment throughout an individual's development. So far, several critical life phases could be identified which are particularly sensitive to alterations in the environment (Hubel and Wiesel, 1970). The impact of social experiences, encountered during these developmental stages, is reflected by changes in neuronal plasticity and distributed brain networks. Hereby, the individuals' behavior is predefined for further encounters throughout adulthood (Pohl et al., 2007). In contrast to the allostatic load hypothesis, the more recent match/mismatch hypothesis of psychiatric disease includes this aspect. It states that early environmental cues influence the development of a phenotype in a manner that provides optimal adaptation to similar environmental conditions later in life. However, a discrepancy between early and late environment would result in maladaptation (Bateson et al., 2004, 2014; Gluckman et al., 2005a,b, 2007; Belsky and Pluess, 2009; Schmidt, 2011; Ricon et al., 2012; Santarelli et al., 2014).

Results of numerous studies show that positive and especially negative experiences during distinct phases of life, ranging from the prenatal and early postnatal stage, through adolescence to adulthood, do have profound effects on the behavioral phenotype (prenatal phase: Cratty et al., 1995; Seckl, 2004; Kaiser and Sachser, 2005; early postnatal phase: Vallée et al., 1997; Caldji et al., 1998; Meaney, 2001; Gross and Hen, 2004; Heiming et al., 2009; Taylor, 2010; Eiland et al., 2012; Ricon et al., 2012; adolescence: Spear, 2000; Schmidt et al., 2007; McCormick et al., 2008; Sachser et al., 2011, 2013; Chaby et al., 2015; Meyer et al., 2016; adulthood: Buwalda et al., 2005; Jansen et al., 2010). However, only little is known about a possible interplay between these developmental stages. Thus, incorporating an experimental design, which combines several socio-environmental interactions during critical life stages throughout lifetime, is needed to outline more natural conditions. A first promising whole life approach was conducted in male mice varying in serotonin transporter (5-HTT) genotype to elucidate the effects of genotype and social environment as well as their interaction on the adult behavioral phenotype. In this study, it has been shown that life history indeed modulates the anxiety-like behavior profoundly (Bodden et al., 2015). More precisely, 5-HTT knockout and wildtype mice that experienced early beneficial and later escapable adverse conditions showed less anxiety-like behavior compared to mice of other life histories. However, it has to be determined how these over lifetime acquired behavioral profiles not only enhance or reduce the expression of anxiety-related behaviors, but especially how they shape fear memory and extinction in adulthood. Since mechanisms underlying fear extinction have attracted considerable interest because of their potential clinical significance, extensive studies have been conducted to understand the importance of single life stages for fear memory and extinction processes in adult humans and rodents (Quirk and Beer, 2006; Myers and Davis, 2007; Sehlmeyer et al., 2009; Lee et al., 2011; Narayanan et al., 2011; Bingham et al., 2013; Shechner et al., 2014; Ponchio et al., 2015; Zoicas and Neumann, 2016).

The aim of the present study was to investigate the effects of either consistent or changing social life experiences on body weight development, anxiety-like behavior, and exploratory locomotion as well as conditioned fear and extinction in adult C57BL/6J mice.

## Materials and methods

All procedures complied with the regulations covering animal experimentation within the EU [European Communities Council DIRECTIVE 2010/63/EU and in accordance with national and local authorities LANUV NRW (reference numbers: 8.87-51.04.20.09.334 and 84-02.05.20.12.212)].

### Animals and housing conditions

All experiments were performed in male C57BL/6J mice, which were bred in the Department of Behavioral Biology at the University of Münster, Germany. The original breeding stock originated from Charles River Laboratories (Sulzfeld, Germany). All animals were housed in transparent standard Makrolon cages type III (38 × 22 × 15 cm) with sawdust and a paper towel as bedding material (Allspan, Höveler GmbH & Co. KG, Langenfeld, Germany), food (1324, Altromin GmbH, Lage, Germany) and water provided *ad libitum* in a temperature and humidity controlled animal facility under a 12 h/12 h light/dark cycle (light on at 8.00 a.m.).

### Experimental design

#### Induction of life histories

**Figure 1:**
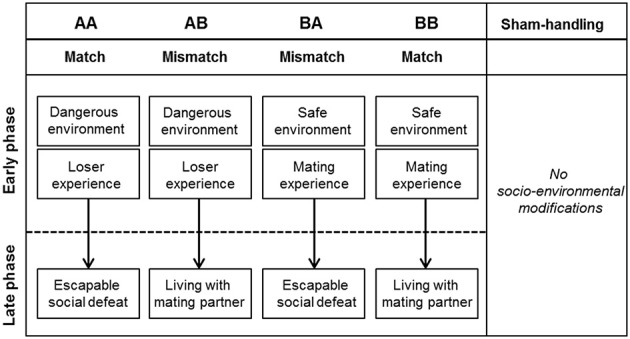
**Overview of the four different life histories and sham-handled mice**. The figure shows the five different experimental groups of male C57BL/6J mice, subdivided into four different life histories (AA, AB, BA, and BB) and a sham-handled group (SH). Every life history comprises an early and a late phase, each being either adverse or beneficial. Sham-handled mice received no modifications in their social environment (*n* = 138) (figure modified after Bodden et al., 2015).

Four different life histories (group size AA: 28, AB: 27, BA: 28, BB: 27) were experimentally induced according to the paradigm published earlier (Bodden et al., 2015). Deviating from the procedure previously described, an additional sham-handled control group was included (group size SH: 28; see Figure 1). Briefly, life histories were divided into an early and a late phase, in which mice were provided with either adverse (“A”) or beneficial (“B”) environmental modifications, or a sham-handling procedure (“SH”). Thus, animals underwent either consistent or changing social life experiences from prenatal stage till adulthood.

In the course of the early phase, two groups (AA and AB group) were exposed to an adverse social environment. For this purpose, pregnant and lactating female C57BL/6J mice were treated with soiled bedding from unfamiliar males. This treatment signals the danger of infant killing and has been shown to cause a significantly higher increase in the females' stress level (e.g., corticosterone) as well as reduced maternal care behavior compared to neutral bedding (Heiming et al., 2009, 2011). On postnatal day (PND) 22, the offspring were separated from the mother and housed in same-sex groups of two to five animals until age of PND 35 ± 2. Subsequently, male mice were single housed in order to exclude social influences such as social hierarchies and agonistic interactions when reunited after loser experience. Yet, due to possible adverse effects, social isolation has to be considered as an experimental limitation of this study. From then on, during adolescence, male offspring of AA and AB group were repeatedly confronted with an adult male mouse from the aggressive NMRI strain (Navarro, 1997) to provide loser experiences. Loser experience increases stress levels and anxiety-like behavior, and thus is considered aversive (5 times, 10 min duration, PND 37 ± 2 – 61 ± 2, Jansen et al., 2010; Kloke et al., 2011). In contrast, two other groups (BB and BA group) experienced beneficial conditions during their early life phase. In this case, female C57BL/6J mice were treated with neutral bedding during pregnancy and lactation to generate a safe environmental setting. With the onset of adolescence, male offspring of BB and BA group repeatedly encountered a female mouse in estrous, allowing for the benefit of mating experiences (5 times, 10 min duration, PND 37 ± 2 – 61 ± 2).

In the course of adulthood (PND 70–77), late phase environmental modifications provided either an adverse or beneficial social experience. For animals of groups AA and BA, an escapable adverse late environment was achieved by confronting C57BL/6J test subjects with a dominant male from the aggressive NMRI strain once a day for the duration of 1 week. These confrontations took place in a custom-made cage system, which provided the C57BL/6J test subject the opportunity to escape to its connected home cage (Lewejohann et al., 2010). This experience has been shown to elicit a significant increase in stress levels in the subdominant animals, and is thus considered an adverse condition (Bodden et al., 2015). In the beneficial social experience design, animals of groups AB and BB gained permanent access to a mating partner for 1 week.

Animals of SH group did not experience any environmental modifications in their life history (neutral environment). Instead of social encounters during adolescence and adulthood, these animals were handled by the experimenter at corresponding times.

After successful generation of the individuals' life history, all experimental subjects were tested for their anxiety-like and exploratory behavior. Subsequently (between PND 77 ± 2 and 82 ± 2), a subset of 75 animals was transported from the Department of Behavioral Biology to the Institute of Physiology 1 (transportation time ~8–10 min), where they were allowed to adapt to the facility for 2 weeks before being fear conditioned and tested for fear memory retrieval, extinction learning and recall of fear extinction as described below.

### Body weights

Animals were weighed during the early phase (PND 22), right before the beginning of the late phase (PND 70 ± 2), and at the end of the late phase (PND 77 ± 2).

### Tests for anxiety-like behavior and exploratory locomotion

Behavioral testing was performed between PND 75 ± 2 and 77 ± 2 as described earlier (Bodden et al., 2015). In total, 138 males (group size: AA: 28, AB: 27, BA: 28, BB: 27, SH: 28) were investigated for their anxiety-like and exploratory behavior in the following order of tests: Elevated plus-maze test (EPM), Dark-light test (DL), and Open field test (OF), with a 24 h delay between each test. All behavioral tests lasted 5 min and were performed in a room different from the housing room during the light phase. Test equipment was cleaned with 70% ethanol between subjects. The animal's movements were recorded by a webcam (Logitech Webcam Pro 9000) and analyzed by the video tracking software ANY-maze (Version 4.75, Stoelting Co., Wood Dale, USA).

Mice were tested in the EPM (Pellow et al., 1985; Lister, 1987, 1990) on PND 75 ± 2. The plus-shaped apparatus, elevated 50 cm above the ground, consisted of two opposing open arms (30 × 5 cm) and two opposing closed arms (30 × 5 cm) with 20 cm high walls that extended from a central square (5 × 5 cm), and was illuminated by a light bulb (150 lx). Parameters analyzed were percentage of time spent on open arms as well as the percentage of entries into open arms to assess anxiety-like behavior, and sum of entries into open and closed arms as an indicator of exploratory locomotion. On PND 76 ± 2, mice were tested in the DL (Crawley and Goodwin, 1980). The apparatus was a modified Makrolon cage type III, which was divided into two compartments by a partition including a sliding door. The light compartment (28 × 27 × 16 cm) had transparent walls, no lid, and was illuminated by overhead lighting (570 lux), while the dark compartment (17 × 27 × 16 cm) was painted black, had an opaque lid, and was unlit. The parameters measured were latency to enter the light compartment and time spent in the light compartment as indicators of anxiety-like behavior, and number of entries into the light compartment to assess exploratory locomotion. The OF (Archer, 1973; Treit and Fundytus, 1988) was performed on PND 77 ± 2 and consisted of a white square arena (80 × 80 × 42 cm), illuminated by an overhead bulb (600 lx). The parameters analyzed were time spent in the center of the arena to measure anxiety-like behavior and distance traveled for assessing exploratory locomotion.

### Fear conditioning and fear memory testing

**Figure 2:**
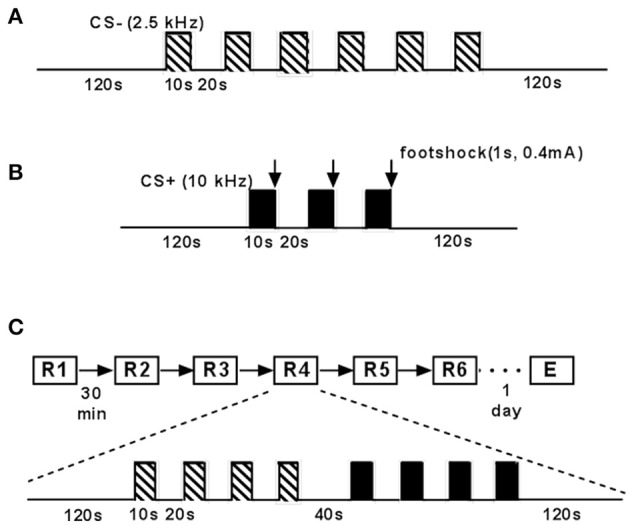
**Schematic drawing of experimental paradigm of fear conditioning and extinction. (A)** Mice were placed in the fear conditioning chamber (context A) and adapted twice (inter-trial interval: 6 h) to a neutral tone (neutral conditioned stimulus, CS−; 2.5 kHz, 85 dB). CS−, indicated by striped bars, was presented 6 times for 10 s with an interval of 20 s. **(B)** C57BL/6J mice underwent fear conditioning in context **(A)**. Conditioned stimulus (CS+; 10 kHz, 85 dB, 10 s), indicated by black bars, was presented 3 times (interval: 30 s) followed by a 0.4 mA electrical stimulus (1 s) indicated by arrows. This was performed twice with an inter-trial interval of 6 h. **(C)** Animals were placed into the recording chamber (context B). After 120 s CS− was presented 4 times (interval: 30 s) followed by 4 CS+ presentations (interval: 30 s) after 40 s. This was performed 6 times (extinction training; inter-session interval: 30 min). Twenty four hours later fear extinction recall was tested with one final repetition.

C57BL/6J mice (*n* = 75) were tested for fear memory retrieval, extinction and extinction recall as published earlier (Seidenbecher et al., 2003; Sangha et al., 2009; Lesting et al., 2011a,b, 2013; Figure 2). Briefly, animals were placed in the fear conditioning chamber (context A) and adapted twice (inter-trial interval: 6 h) to a neutral tone (CS−: 2.5 kHz, 85 dB SPL). CS− was presented 6 times for 10 s with an interval of 20 s (Figure 2A). On the second day, mice underwent fear conditioning in the fear conditioning chamber (context A). The conditioned stimulus (CS+, 10 kHz, 85 dB SPL, for 10 s) was presented 3 times with an inter-stimulus interval of 20 s and co-terminated for 1 s with a 0.4 mA unconditioned stimulus (footshock). This session was performed twice with an inter-trial interval of 6 h (Figure 2B). On the following day, animals were placed into an open-field like arena (neutral context, context B), made up by a standard makrolon cage type III. In a first retrieval session (R1), CS− was presented 4 times (10 s, ISI 20 s) followed by 4 CS+ presentations (10 s, ISI 20 s) after 40 s. This session was repeated 5 times (R2–R6) with an inter-session interval of 30 min, representing extinction learning. Fear extinction recall (E) was tested on day 4. In this case animals underwent the same procedure as on day 3 (Figure 2C).

### Behavioral expressions after fear conditioning

To detect discrete behavioral changes after fear conditioning, the following behavioral expressions were assessed online via visual observation by an experimenter blind to the life history: freezing, risk-assessment, flight, grooming, exploration, rearing, and quiescence. Freezing was defined as absence of movement except respiratory movements and attentive watching (Grossen and Kelley, 1972; Fanselow and Bolles, 1979; Blanchard et al., 1997; Gerlai, 1998; Laxmi et al., 2003). Risk-assessment was scored, if animals displayed stretched-attention and sensory scanning (sniffing, auditory, and visual scanning, marked by side-to-side head sweeps) (Blanchard et al., 2003). Flight was rated, if animals reacted with sudden running, followed by freezing behavior. Grooming was assessed during phases of mouth washing, fur and paw licking and whisker cleaning. Exploration was defined as animals in motion (walking and running). Rearing was rated, if animals positioned themselves on hind limbs. Quiet was rated, if animals were in rest, but not sleeping and showed no further movement except respiratory movement and no further reaction toward tone presentation.

For the determination of life history-dependent changes in total defense, comprising freezing, risk-assessment and flight (Laxmi et al., 2003), analysis was performed for percentage of time within 40 s bins for time periods preCS− (40 s prior first CS onset), CS− (total of 4 CS− presentations), preCS+ (40 s interval between last CS− offset and first CS+ onset), CS+ (total of 4 CS+ presentations) and postCS+ (40 s after last CS+ offset).

To assess the impact of life histories on the individual behavioral profile (“discrete behavioral expressions”) comprising the behavioral expressions as described above each behavior, was analyzed separately as percentage of time for preCS−, CS−, preCS+, CS+, and postCS+. Importantly, for both analyses (total defense and discrete behavioral expressions) preCS− of R1 was considered as “behavioral baseline,” since during this time period animals were placed in a novel, open field-like context.

Furthermore, to elucidate effects of life history on fear retrieval and extinction of conditioned fear, freezing behavior was analyzed for 10 s of first CS− and CS+ presentation (firstCS− and firstCS+) as percentage of time spend freezing during retrieval and extinction sessions as published before (Sangha et al., 2009; Lesting et al., 2011a,b, 2013).

### Statistics

General Linear Models (GLM) were used for the analysis of the obtained data. In case of significant main or interaction effects, *post-hoc* pairwise comparisons of different levels were conducted using Bonferroni adjustment. All data are expressed as mean ± SEM. Statistical significance for all experiments was *p* < 0.05 (^*^), *p* < 0.01 (^**^), and *p* < 0.001 (^***^). Statistical analyses were conducted using the statistical software IBM SPSS Statistics (IBM Version 23, Release 2015) and STATISTICA 12 (StatsoftEurope, Release 2012). Graphs were created using the software GraphPad Prism 6 for Windows (GraphPad Software, Inc., Release 2014).

#### Body weights and anxiety-like behavior

GLM were applied for the analysis of body weights and data obtained from tests for anxiety-like behavior and exploratory locomotion. In order to meet the assumptions of parametric analysis, residuals were graphically examined for homoscedasticity and outliers and the Lilliefors corrected Kolmogorov-Smirnov Test was applied. When necessary, raw data were transformed using either logarithmic or square root transformations.

In particular, ANOVA with repeated measures was performed for the analysis of body weight with within-subjects factor “time” (postnatal day, PND), fixed between-subject factor “life history” and the interaction of “life history” and “time.”

Univariate ANOVA was used to analyze several dependent variables (anxiety-like and exploratory behaviors) with fixed between-subject factor “life history.”

For the analysis of “early vs. late phase” effects (without group SH), ANOVA was performed for several dependent variables (body weights, anxiety-like and exploratory behaviors) with fixed between-subject factors “early phase” (adverse: AA and AB; beneficial: BA and BB), “late phase” (adverse: AA and BA; beneficial: AB and BB), and the interaction of “early phase” and “late phase.”

#### Total defense and behavioral expressions of conditioned fear

ANOVA with repeated measures with first within-subject factor “session” (R1, R6, and E), second within-subject factor “time periods” (preCS−, CS−, preCS+, CS+, and postCS+), and between-subject factor “life history” was used to analyze total defense and individual behaviors, such as freezing, risk-assessment, rearing, flight, exploration, grooming and quiet, of life history dependent behavioral expressions.

To determine the “behavioral baseline” after fear conditioning, ANOVA with repeated measurements with R1, R6 and E as within-session factors and “life history” as between-subject factor was performed for total defense and discrete behavioral analysis during preCS− in R1.

To determine dynamics of fear retrieval and extinction, ANOVA with repeated measures was utilized, with freezing within 10 s bins (firstCS− and firstCS+) as within-subjects factor during sessions and as between-subjects factor for “life history.”

To detect “early vs. late” phase effects of life history (without group) on the different analyzed parameters, multifactorial ANOVA with repeated measures was performed with “session” (R1, R6, and E) as first within-subject factor, “time periods” (preCS−, CS−, preCS+, CS+, and postCS+) as second within-subject factor and “early phase” (adverse: AA and AB; beneficial: BA and BB) and “late phase” (adverse: AA and BA; beneficial: AB and BB) as between-subject factors for total defense, behaviors (40 s bins) and freezing (10 s bins).

## Results

Four different life histories were induced in male C57BL/6J mice comprising an “early phase” and “late phase” which were either dedicated by an adverse (A) or beneficial (B) social environment. Consequently, animals of a matched life history were represented by groups AA (adverse early and late phase) and BB (beneficial early and late phase) while mismatched life histories were introduced to mice of groups AB (adverse early phase and beneficial late phase) and BA (beneficial early phase and adverse late phase). A fifth group, that was solely sham-handled after separation from the litter, served as control for possible handling effects (SH) (Figure 1). Over the course of each life history (PND 22, 70 ± 2, and 77 ± 2) body weights were measured and in addition animals were tested in three tests for anxiety-like behavior and exploratory locomotion (PND 75 ± 2 − 77 ± 2). Subsequently, animals were trained in a Pavlovian fear conditioning paradigm.

### Body weight

A significant main effect of life history on body weight development was detected [*F*_(4, 133)_ = 3.373, *p* < 0.05]. Furthermore, the “early vs. late phase” analysis revealed a significant main effect of the early phase on body weights PND 22: [*F*_(1, 106)_ = 4.186, *p* < 0.05], PND 70 ± 2: [*F*_(1, 106)_ = 17.691, *p* < 0.001]; PND 77 ± 2: [*F*_(1, 106)_ = 7.435, *p* < 0.01]. It was found that adversity during the prenatal and early postnatal phase caused higher body weights compared to a safe environment (PND 22). Additionally, body weights in mice experiencing adversity during adolescence were increased in comparison to mice in the beneficial condition (PND 70 ± 2). This effect lasted into adulthood (PND 77 ± 2). For a detailed description of the results, see Appendix.

### Anxiety-like behavior and exploratory locomotion

**Figure 3:**
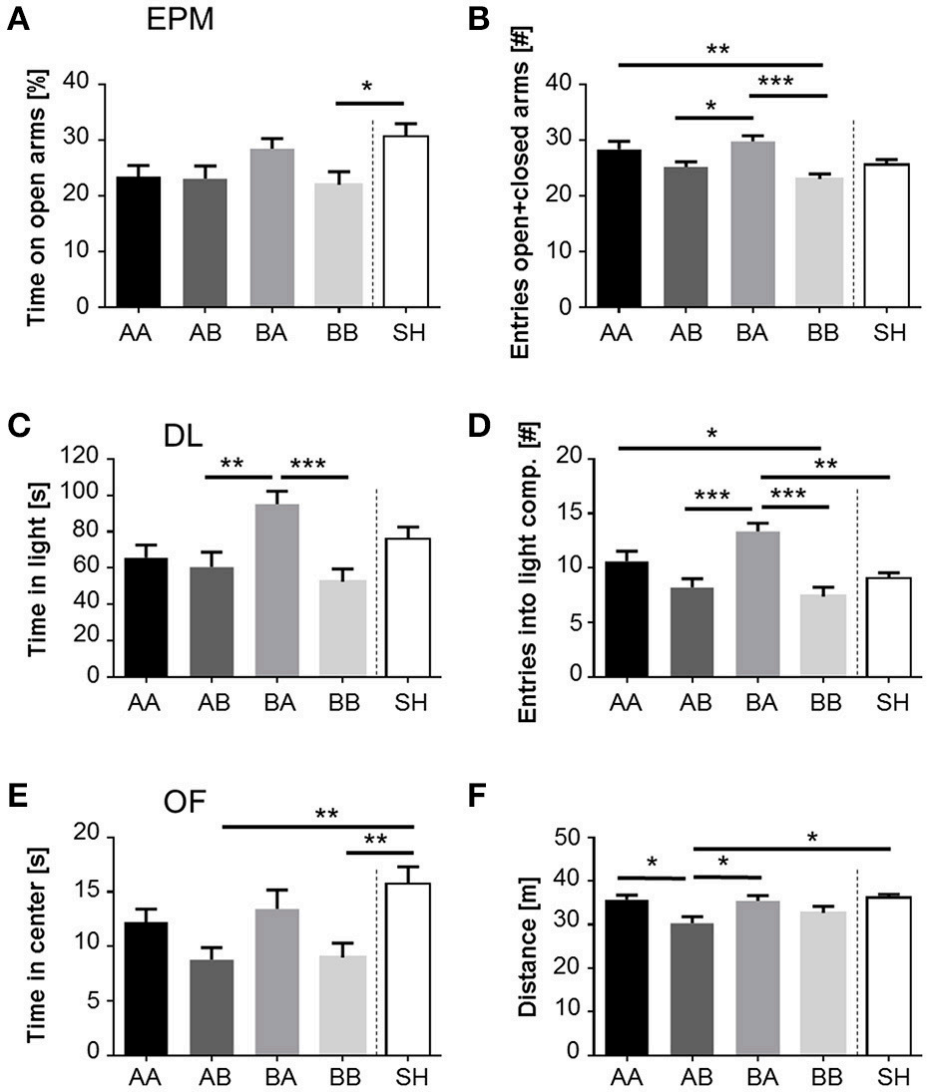
**Impact of life history on anxiety-like behavior and exploration. (A)** Time spent on open arms of EPM, **(B)** Sum of entries into open and closed arms of EPM, **(C)**, Time spent in and **(D)** Entries into light compartment of DL, **(E)** Time spent in center and **(F)** Distance traveled in OF, displayed by male mice grown up in an early adverse (AA and AB) or beneficial (BA and BB) environment and provided with later matching (AA and BB) or mismatching (AB and BA) living conditions in adulthood. Data are given as means +SEM. Statistics: ANOVA; *post-hoc* testing: Bonferroni. ^*^p < 0.05; ^**^p < 0.01; ^***^p < 0.001. Animals per group: AA = 28, AB = 27, BA = 28, BB = 27, SH = 28.

**Figure 4:**
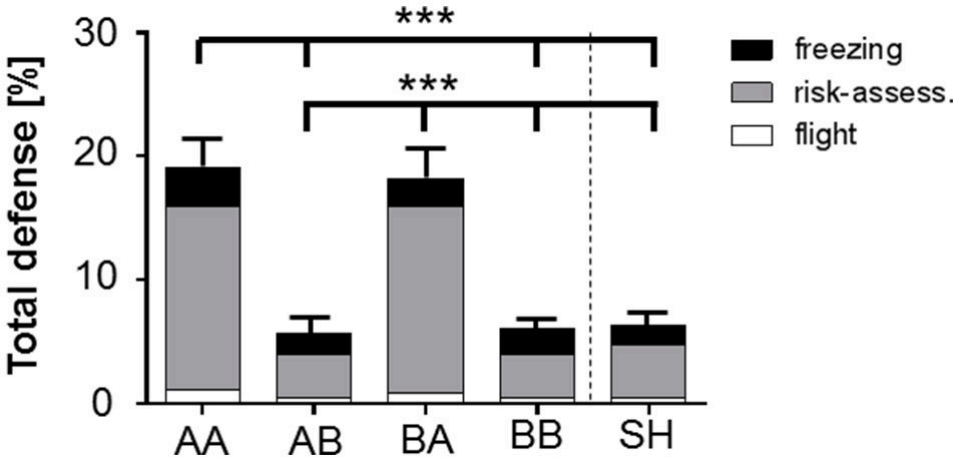
**Stacked bar graph illustrates the impact of life history on total defensive behavior (freezing, risk-assessment, flight) during behavioral baseline (preCS− in R1)**. Total defense (freezing, risk-assessment, flight; % of time) scored for behavioral baseline (preCS− in R1) showed a significant increase for AA and BA animals compared to AB, BB, and SH life histories prior first stimulus presentation. Analysis of proportional values for individual behaviors revealed that this effect was due to a significant increase in risk-assessment. Life histories: AA, early and late adversity; AB, early adversity and late benefit; BA, early benefit and late adversity; BB, early and late benefit; SH, sham-handled. ^***^p < 0.001; animals per group: *n* = 15.

Anxiety-like behavior was significantly influenced by life history, as demonstrated by the percentage of time on the open arms of the EPM [*F*_(4, 133)_ = 3.237, *p* < 0.05], the time spent in the light compartment of the DL [*F*_(4, 133)_ = 5.708, *p* < 0.001], and the time spent in the center of the OF [*F*_(4, 133)_ = 4.894, *p* < 0.01; Figures 3A,C,E]. *Post-hoc* analysis revealed that BA mice spent significantly more time in the light compartment than AB (*p* < 0.01) and BB mice (Figure 3C; *p* < 0.001). Furthermore, SH mice spent significantly more time on the open arms than BB mice (Figure 3A; *p* < 0.05) and remained longer in the center of the OF compared to AB and BB mice (Figure 3E; *p* < 0.01). Neither the percentage of entries into open arms of the EPM nor the latency to enter the light compartment of the DL was significantly influenced by life history. In summary, life history significantly influenced anxiety-like behavior. Particularly low levels of anxiety were found in animals experiencing early beneficial and later adverse conditions (BA) as well as in sham-handled individuals (SH). Concerning exploratory locomotion, significant main effects of life history were found on the sum of entries into open and closed arms of the EPM [*F*_(4, 133)_ = 6.178, *p* < 0.001], number of entries into the light compartment of the DL [*F*_(4, 133)_ = 9.069, *p* < 0.001], and distance traveled in the OF [*F*_(4, 133)_ = 4.081, *p* < 0.01; Figures 3B,D,F]. *Post-hoc* testing revealed that the sum of entries into open and closed arms of the EPM as well as the number of entries into the light compartment of the DL was higher in BA mice compared to both AB (EPM: *p* < 0.05; DL: *p* < 0.001) and BB mice (EPM, DL: *p* < 0.001; Figures 3B,D). BA mice entered the light compartment more frequently than SH mice (*p* < 0.01) and traveled significantly more in the OF than AB mice (*p* < 0.05; Figure 3F). AA mice showed a higher sum of entries into open and closed arms (Figure 4B; *p* < 0.01) and more entries into the light compartment than BB mice (Figure 3D; *p* < 0.05). AA as well as SH mice traveled significantly more in the OF than AB mice (Figure 3F; AA vs. AB, SH vs. AB; *p* < 0.05). In summary, life history significantly influenced exploratory locomotion. In particular, animals experiencing early beneficial and later adverse conditions (BA) exhibited highest levels of exploratory locomotion.

The additional “early vs. late phase” analysis revealed a significant main effect of the late phase on both anxiety-like behavior [time in light compartment of DL: *F*_(1, 106)_ = 11.977, *p* < 0.001; latency to enter light compartment of DL: *F*_(1, 106)_ = 5.253, *p* < 0.05; time in center of OF: *F*_(1, 106)_ = 9.000, *p* < 0.01] and exploratory locomotion [sum of entries in EPM: *F*_(1, 106)_ = 19.939, *p* < 0.001; entries into light compartment of DL: *F*_(1, 106)_ = 25.003, *p* < 0.001; distance traveled in OF: *F*_(1, 106)_ = 9.460, *p* < 0.01]. Escapable adversity in adulthood (AA, BA) was linked to significantly lower levels of anxiety and higher levels of exploratory behavior. Moreover, a significant early-by-late phase interaction was detected regarding measures of anxiety-like behavior [time in light compartment of DL: *F*_(1, 106)_ = 5.445, *p* < 0.05] and exploratory locomotion [entries into light compartment of DL: *F*_(1, 106)_ = 4.628, *p* < 0.05]. *Post-hoc* analysis demonstrated that early beneficial conditions caused significantly lower levels of anxiety-like behavior in mice experiencing later adverse conditions (BA) compared to later beneficial conditions (BB, time in light compartment: *p* < 0.001). Moreover, mice that grew up under early beneficial conditions exhibited significantly higher levels of exploratory locomotion when confronted with adverse (BA) compared to beneficial conditions (BB) in adulthood (entries into light compartment: *p* < 0.001). Additionally, later life adversity caused significantly increased exploratory behavior in mice experiencing early life benefits (BA) compared to early life adversity (AA, entries into light compartment: *p* < 0.05).

### Behavioral expressions of conditioned fear

After the completion of the life history paradigm, animals were trained in a Pavlovian fear conditioning paradigm (Figure 2) to address the question how an individual's whole life experience influences not only the behavioral profile, but processes of conditioned, retrieved, and extinguished fear in particular.

In a first approach we cumulated freezing, flight, and risk-assessment as total defense to detect major defensive behavioral changes between sessions and groups.

#### Total defense at behavioral baseline

Evaluation of baseline behavior was performed for time period preCS− during retrieval of learned fear (R1), in which animals were exposed to the novel, open-field-like context. Analysis of total defense during “behavioral baseline” ANOVA showed a main effect of life history [*F*_(4, 70)_ = 13.841, *p* < 0.001]. Bonferroni *post-hoc* analysis revealed that animals of group AA and BA showed significantly increased defensive behaviors even prior to the first sessions' first neutral stimulus presentation (preCS−) during retrieval (R1) (Figure 4, Table 1; AA vs. AB, BB and SH: *p* < 0.001; BA vs. AB, BB and SH: *p* < 0.001).

**Table 1:** **Influence of life history on total defensive behavior (freezing, risk-assessment, flight)**.

	**preCS−**					**CS−**					**preCS+**					**CS+**					**postCS+**				
	**AA**	**AB**	**BA**	**BB**	**SH**	**AA**	**AB**	**BA**	**BB**	**SH**	**AA**	**AB**	**BA**	**BB**	**SH**	**AA**	**AB**	**BA**	**BB**	**SH**	**AA**	**AB**	**BA**	**BB**	**SH**
R1	19.2	5.7	18.2	6.0	6.4	77.1	61.3	66.8	66.7	69.1	38.4	16.5	30.3	17.0	16.8	90.5	87.0	88.7	89.8	88.6	30.6	17.2	25.1	9.5	15.3
R6	17.9	9.9	20.5	2.7	6.2	37.5	19.3	30.4	6.9	13.7	24.6	13.1	15.7	8.4	14.5	71.7	22.4	58.4	18.4	26.3	19.6	5.8	12.9	6.3	5.4
E	17.1	11.4	12.4	5.1	8.9	45.4	30.2	36.7	23.8	35.4	27.3	17.6	22.3	13.1	12.3	84.9	76.5	69.2	60.9	55.3	16.9	12.7	11.5	9.0	13.9

*Life histories: AA, early and late adversity; AB, early adversity and late benefit; BA, early benefit and late adversity; BB, early and late benefit; SH, sham-handled. Fear memory states: R1, retrieval session 1; R6, retrieval session 6 (end of extinction training); E, extinction recall. Total defensive behavior (% of time) was scored before, pre and post CS− and CS+ presentation, respectively. Animals per group: n = 15*.

Additional analysis of “discrete behavioral expressions” during preCS− in retrieval showed that the impact of total defense was caused by an increased display of risk-assessment. ANOVA with risk-assessment as dependent variable detected a main effect of life history during preCS− [*F*_(4, 70)_ = 11.93, *p* < 0.001]. *Post-hoc* analysis revealed that already prior to first stimulus presentation animals of group AA and BA displayed significantly elevated levels of risk-assessment when compared to other groups (Figure 4; *p* < 0.001). No significant effects could be determined for freezing, flight, grooming, exploration, rearing and quiet during retrieval baseline (Supplementary Figure S2, Supplementary Table S1).

#### Total defense in response to stimulus presentation

**Figure 5:**
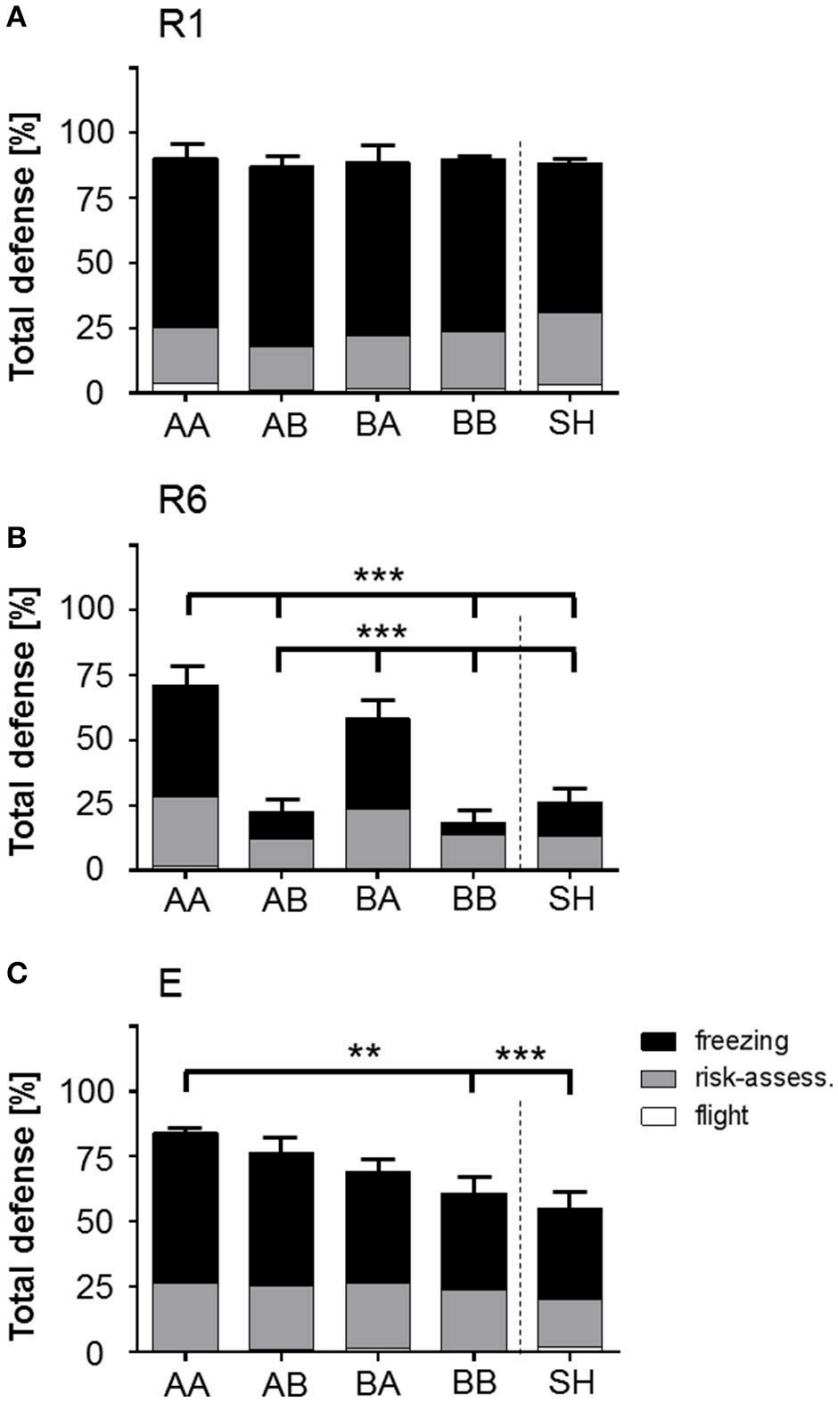
**Stacked bar graphs illustrate the expression of total defensive behaviors in response to CS+ presentation during fear retrieval (R1), extinction (R6), and extinction recall (E)**. Total defense (TD; freezing, risk-assessment, flight; % of time) scored for mean of all four CS+ presentations during R1, R6, and E showed that **(A)** during R1, all groups similarly expressed defensive behaviors upon stimulus presentation. **(B)** R6: At the end of extinction training, AA and BA animals showed significantly elevated levels of TD in comparison to other life histories, which were mainly a result of significantly prolonged freezing periods upon stimulus presentation. **(C)** E: AA animals showed significantly increased TD in comparison to BB and SH animals due to elevated freezing. Life histories: AA, early and late adversity; AB, early adversity and late benefit; BA, early benefit and late adversity; BB, early and late benefit; SH, sham-handled. ^**^p < 0.01; ^***^p < 0.001; animals per group: *n* = 15.

Analysis focused on changes in total defense and discrete behavioral expressions during all four CS+ presentations during retrieval, extinction learning and extinction recall (Figure 5, Table 1, Supplementary Figure S2, Supplementary Table S1).

Here, analysis revealed a significant interaction effect of time period × session × life history on total defense [*F*_(32, 560)_ = 4.442, *p* < 0.001]. Evaluation of total defense revealed that proportional values of expressed behaviors showed an ubiquitous increase of defensive behaviors upon CS+ presentations in all groups over subsequent sessions. Bonferroni *post-hoc* analysis on interaction between time period × session × life history for between-group comparisons showed no significant effects for retrieval, but analysis of extinction learning revealed that during CS+ presentation AA and BA animals showed a significant elevation in total defense as compared to AB, BB, and SH groups (Figure 5; *p* < 0.001). During extinction recall this effect was solely observed in AA mice as compared to BB (*p* < 0.01) and SH (*p* < 0.001).

Additional “early vs. late phase” analysis revealed an interaction effect of session × time period × late phase on total defense [*F*_(8, 448)_ = 1787.0, *p* < 0.001]. *Post-hoc* analysis demonstrated that, as compared to late life benefit (AB and BB), escapable adversity in adulthood (AA and BA) was correlated with significant elevation in total defense during CS− and CS+ presentation at the end of extinction training (R6: CS− and CS+, *p* < 0.001).

Regarding discrete behavioral expressions of total defense, between-group comparison during CS+ presentation revealed significant elevation of freezing duration for AA and BA animals as compared to AB, BB, and SH groups in extinction learning (R6; *p* < 0.001). At extinction recall (E) a significant increase in time spent freezing was observed for AA animals as compared to BB, BA, and SH mice (*p* < 0.001) and AB group as compared to BB (*p* < 0.01) and SH (*p* < 0.001) animals. Analysis of risk-assessment and flight as further behavioral components of total defense revealed no statistical significance for CS+ time period.

Analysis of “early vs. late phase” for freezing duration revealed a significant interaction effect of session × time period × early phase [*F*_(8, 448)_ = 3.678, *p* < 0.001] and session × time period × late phase [*F*_(8, 448)_ = 14.645, *p* < 0.001]. *Post-hoc* analysis showed that an adverse early life (AA and AB) significantly increased freezing duration during CS+ at extinction learning (R6; *p* < 0.01) and at extinction recall (E; *p* < 0.001) as compared to a beneficial early life (BA and BB). Also, adversity experienced in the late phase (AA and BA) significantly increased freezing in response to CS+ at extinction learning (*p* < 0.001) as compared to late life benefit (AB and BB). Detailed analysis of all discrete behavioral expressions are described in the Appendix.

In a brief summary, AA animals displayed highest levels of total defense in response to the conditioned stimulus which were maintained throughout retrieval (R1), extinction learning (R6) and extinction recall (E) as compared to other groups. Additionally, AA mice showed increased defensive behaviors during CS− at R1. Interestingly, AA and BA animals showed defensive behaviors already prior to the first stimulus presentation (preCS−) during retrieval.

Next, analysis of freezing behavior during presentation of the first CS+ (duration 10 s) of each session was considered separately for mice of each life history group.

#### Freezing (firstCS+)

**Figure 6:**
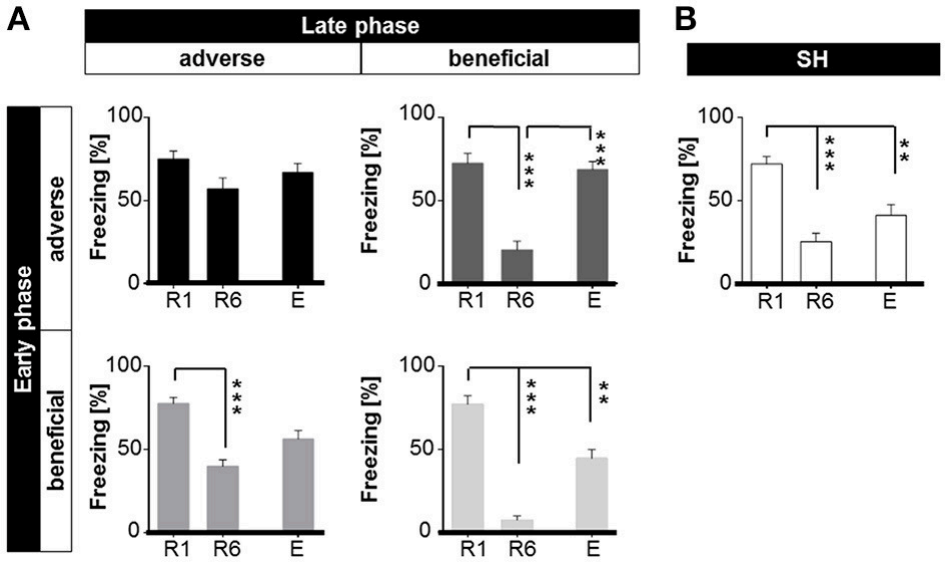
**Expression of freezing (% of time) in response to first CS+ presentation during retrieval (R1), extinction (R6), and extinction recall (E) dependent on different life histories. (A)** Life histories: early and late adversity (AA, top left) mice showed no decline in freezing. Early and late benefit (BB, low right) was significantly accompanied with the capability of extinction learning (R1 to R6) and extinction memory consolidation (R1 to E). Mismatched life history mice (AB and BA, top right and low left) extinguished learned fear, though consolidation of extinction memory (E) was affected. **(B)** Sham-handled animals significantly extinguished learned fear and consolidated extinction memory. ^**^p < 0.01; ^***^p < 0.001; animals per group: *n* = 15.

ANOVA with repeated measures with session (R1-E) as dependent variable and group as independent variable revealed a significant interaction of session × life history [*F*_(8, 140)_ = 5.107, *p* < 0.001]. Bonferroni *post-hoc* analysis showed that AA animals displayed a pronounced level of freezing upon CS+ presentation during retrieval (R1), extinction learning (R6), and extinction recall (E) (Figure 6A). AB mice that were initially raised under adverse social conditions and subsequently encountered a beneficial environment showed a significant reduction of freezing behavior (*p* < 0.001) in retrieval session R6. However, when tested for extinction recall, AB mice exhibited a significant increase in freezing duration during extinction recall (*p* < 0.001; Figure 6A). BA animals successfully diminished freezing duration (*p* < 0.001) after extinction training, but showed no significant change in freezing duration between values for retrieval (R1) vs. extinction recall (E) and extinction learning (R6) vs. extinction recall (E) (Figure 6A). In contrast, *post-hoc* analysis showed that BB and SH mice decreased their freezing response upon CS+ presentation significantly (*p* < 0.001) during extinction learning and extinction recall (*p* < 0.001; Figures 6A,B). Furthermore, mice of all groups showed significantly increased freezing upon CS+ compared to CS− presentation (*p* < 0.001), indicating a cue specific fear response (Figure 6, Supplementary Figure S3).

**Figure 7:**
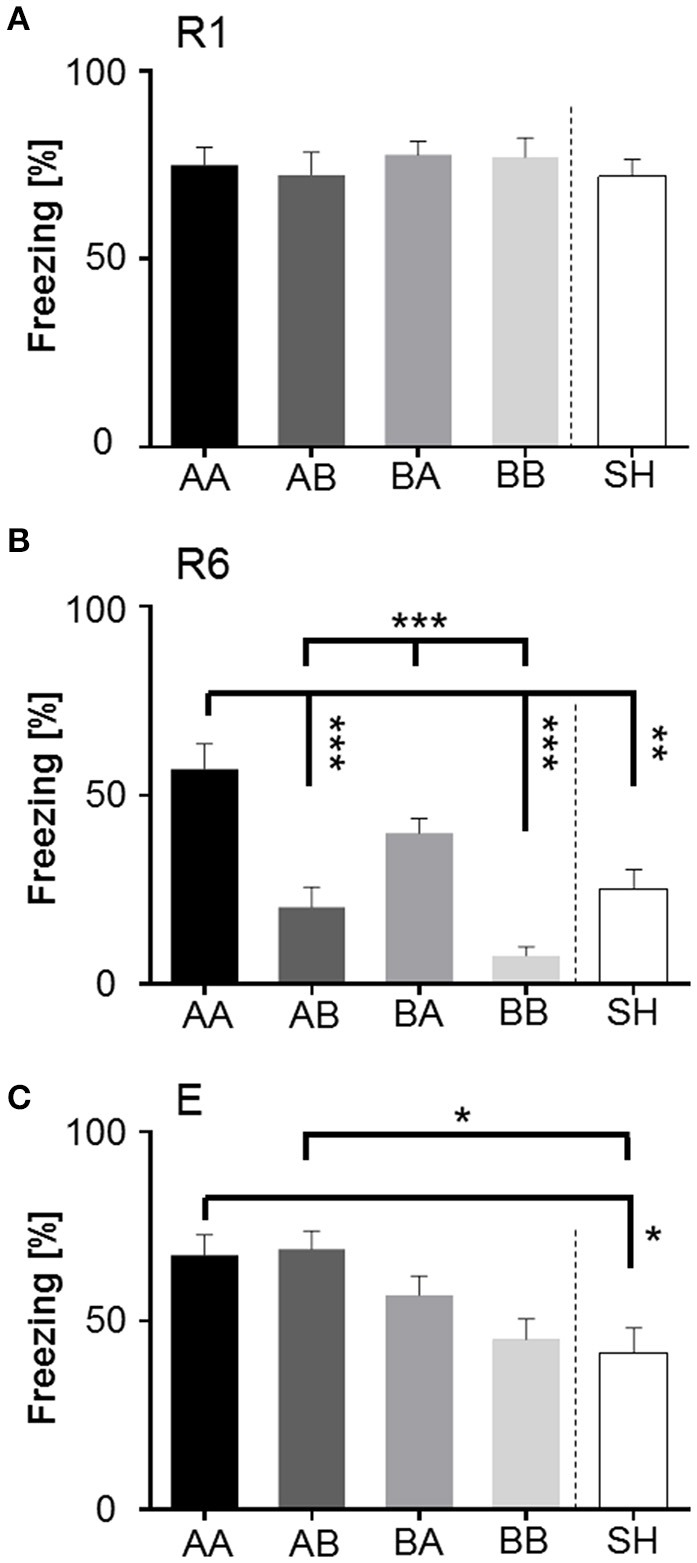
**Between-group comparison of freezing (% of time) in response to first CS+ presentation during fear retrieval (R1), extinction (R6), and extinction recall (E). (A)** R1: No significant differences were detected between groups, implying that all animals acquired conditioned fear memory to the same extent. **(B)** R6: Comparison between groups revealed that matched life history had the strongest diverging effect during fear extinction learning. Early and late adversity (AA) compromised extinction training, while early and late benefit (BB) significantly facilitated extinction learning. Animals experiencing late life adversity (BA) also showed significantly elevated freezing, whereas late life benefit (AB) as well as sham-handled mice (SH) showed moderate levels. **(C)** E: Mice subdued to early life adversity (AA and AB), were significantly negatively affected when tested for extinction memory consolidation in comparison to sham-handled mice. Animals of early life benefit showed moderate expression of freezing in response to CS+. ^*^p < 0.05; ^**^p < 0.01; ^***^p < 0.001; animals per group: *n* = 15.

Bonferroni *post-hoc* analysis between groups showed that all groups exhibited similar freezing levels during retrieval (Figure 7A). Yet significant group-dependent differences could be detected during extinction learning (Figure 7B): AA mice showed significantly augmented freezing duration when compared to AB (*p* < 0.001), BB (*p* < 0.001), and SH mice (*p* < 0.01). Similarly, BA mice exhibited a significant increase in freezing duration upon CS+ presentation in comparison to BB and AB (*p* < 0.001). However, at extinction recall (E), AA, and AB animals displayed significantly increased freezing duration as compared to SH mice (Figure 7C; *p* < 0.05).

“Early vs. late phase” analysis revealed a significant main effect of “early phase” [*F*_(2, 112)_ = 5.231, *p* < 0.01] and “late phase” [*F*_(2, 112)_ = 13.026, *p* < 0.001] on freezing. Bonferroni *post-hoc* analysis revealed that “early” as well as “late” adverse experience increased freezing duration. Especially early life adversity compared to early benefit was related to elevated freezing levels during extinction learning (R6; *p* < 0.001) and extinction recall (E; *p* < 0.01). Late life adversity, as compared to late life benefit, resulted in high freezing during extinction learning (*p* < 0.001).

It can be summarized, that accumulation of adversity (AA) throughout life history led to increased freezing during the first CS+ during retrieval (R1), extinction learning (R6) and extinction recall (E). All groups (except AA) significantly diminished freezing from R1 to R6. At recall of extinction (E), BA, and AB mice showed increased freezing as in contrast to BB and SH animals. It could be shown that late life adversity as compared to late life benefit mainly affected extinction learning (R1–R6), while early life adversity as compared to early life benefit interfered with extinction recall. Furthermore, the experience of late life adversity (AA and BA) resulted in increased risk-assessment during behavioral baseline. Accumulation of adversity in AA animals led to increased flight behavior. In contrast, BB mice were most quiet at the end of extinction training (R6).

## Discussion

Our study focused on how the experience of a matched/mismatched whole life history modulates anxiety-like behavior and fear memory during adulthood in C57BL/6J male mice. The aim was to create a realistic and natural life history for mice, which included a match/mismatch experience. In principle, four phases of life (prenatal and early postnatal phase, adolescence, and adulthood) were manipulated in this approach, which mimics a match/mismatch condition in close analogy to human life histories (McEwen, 2003; Gluckman et al., 2007). Although it is known, that distinct manipulation during each of these life phases can lead to vastly different outcomes (for detailed references see Introduction), we show that the establishment of this specific life history approach has a significant impact on (i) body weight gain, and (ii) anxiety-like behavior and exploratory locomotion. Life history modification also resulted in (iii) divergent, life history-dependent, changes in total defense and discrete behavioral expressions, but (iv) had no effect on fear retrieval. It could further be concluded that (v) the experienced life history shaped fear extinction and extinction consolidation for each life history combination in a distinct manner.

### Impact of life history on body weight gain, anxiety-like behavior, and exploratory locomotion

Life history was found to significantly influence body weights, similar to the previous findings (Bodden et al., 2015). We show that adverse experiences in form of exposure to olfactory cues of unfamiliar adult males during prenatal and suckling period caused significantly higher body weights at the time of weaning (PND 22) compared to a safe early environment in the offspring. This finding indicates the relevance of this stimulus, which simulates a high density of foreign, infanticidal male mice (Heiming et al., 2009, 2011), hereby generating an adverse early life. Similarly, Dantzer et al. (2013) found that offspring of female North American red squirrels, which experienced experimentally heightened perceived population density, gained weight significantly faster than offspring of control females. Furthermore, we found that loser experiences during adolescence led to a significant increase in body weights compared to mating experiences, exactly as it was found in the preceding study (Bodden et al., 2015). Such an association between social defeat and increased body weight has also been found by Bartolomucci et al. (2004) and Nestler (2012).

Anxiety-like behavior and exploratory locomotion were assessed using three standard tests (EPM, DL, and OF). Our data reveal that life history has a profound effect on state anxiety and exploration. Similar to our previous findings (Bodden et al., 2015), we could confirm the finding that a beneficial early life followed by escapable adversity (BA) causes decreased anxiety-like behavior and increased exploratory locomotion in comparison to most other life histories. This result shows that limited adversity in later life can have a positive effect on state anxiety in comparison to both accumulating adversity and sheltering from all stressors. Thus, the experience of some adversity may promote the ability of an individual to better cope with future challenges (Dienstbier, 1989; Fontana and Rosenheck, 1998; Parker et al., 2004, 2006; Seery et al., 2010, 2013; Sachser et al., 2011; Meyer et al., 2016).

### Impact of life history on total defense, discrete behavioral expressions and fear and extinction memory

Freezing, flight and risk-assessment were assessed and cumulated to identify life history-dependent changes in total defensive behaviors in mice. Our data show that levels of total defensive behaviors were identical between groups and balanced throughout time intervals (e.g., preCS−, CS−, etc.) during fear retrieval. All groups were capable of identifying the neutral stimulus as non-threatening cue, as reflected by significantly lowered total defense at the end of extinction training. Mice experienced with whole life adversity (AA) displayed higher levels of arousal, as indicated by a higher percentage of total defensive behavior upon both presented stimuli and only a low decline of defensive actions. This suggests that an accumulation of adversity might not only contribute to higher alertness, but also to an overexpression of underlying defensive behaviors. In line with this finding, a previous study conducted on life history, reported that the generation of AA life history indeed resulted in high levels of anxiety-like behavior and decreased locomotor activity in male 5-HTT mice, which could contribute to heightened defensive behaviors as observed in this study (Bodden et al., 2015). Individual life history analysis reveals that experienced early life adversity and late life benefit (AB) led initially to a decrease in total defense during extinction training, but recovered to high values at extinction recall. Comparison of life history groups and sham-handled mice reveals that chronic subordination during adolescence (AA and BA) results in higher levels of defensive behaviors upon CS− and CS+ presentation. This finding is confirmed by the analysis of “early vs. late phase” for life history mice. Other studies reported that after social defeat stress rodents showed a consistent increase in anxiety in the elevated plus-maze (Korte and de Boer, 2003; Klinsey et al., 2007; Caldwell and Riccio, 2010; Hammels et al., 2015).

Explicit analysis of distinct behavioral expressions allowed a more defined readout of individual behaviors after fear conditioning. In AA mice, accumulation of lifetime adversity did not compromise extinction learning processes in terms of freezing, but might lead to higher states of arousal, as deduced from increased risk-assessment and flight behavior in absence of potential threats. AA mice displayed a high percentage of risk-assessment even in absence of a potential threat during behavioral baseline, and a higher probability to respond with flight upon the presented stimuli compared to other life history groups. Furthermore, although being capable of reducing freezing upon stimuli presentation in the course of extinction training, AA mice showed highest freezing of all groups. McEwen reviewed that accumulation of stress would add to the individuals “allostatic load” and consequently produce cumulative changes in both brain and body (McEwen, 2003). It is suggested that a chronic imbalance in the activity of adaptive systems, such as the hypothalamic-pituitary-adrenal (HPA) axis and endogenous neurotransmitters such as glutamate, would contribute to atypical behavioral coping responses, reflecting emotional arousal and psychic disorganization as one might assume for AA life history mice (Sachar et al., 1970; McEwen, 2003).

In contrast, from our data on mice which experienced a matched beneficial life history (BB), it can be presumed that an overall beneficial social environment facilitates fear learning and memory and allows faster adaption to the relevant cue and the experimental environment. Our data show that BB mice displayed low risk-assessment and flight behavior and a rapid decline of freezing in the course of extinction training. Furthermore, in comparison to all groups, highest levels of quiet were observed, even in the presence of both stimuli at the end of extinction learning (R6), indicating the capability of enhanced emotional adaption. In rodents, it has been shown that high quality of maternal care (Caldji et al., 1998) and mating experience (Edinger and Frye, 2007; Kästner et al., 2015) positively mediate the individuals' expression of fear and anxiety-like behaviors. Therefore, it can be assumed that a combination of these social experiences would contribute to the observed lowered vulnerability of adult BB mice.

Our findings on a mismatched life history in mice, which initially experienced early life adversity, followed by late life benefit (AB), raise the notion that this pairing did not interfere with the individuals' basal behavioral expressions *per se*, but might compromise consolidation processes of long-term emotional memory formation of defensive behaviors. Like BB mice, AB animals displayed low levels of defensive behaviors and reduced freezing in the presence of CS+ during extinction training. Interestingly, although this life history group showed freezing levels in the range of BB and SH group during extinction learning, AB mice responded with a strong elevation of freezing levels upon CS+ presentation also during extinction recall. In fact, prenatal stress in rodents was shown to interfere with adult behavior in terms of attention and learning deficits, increased anxiety-like behavior and depression (Weinstock, 2008; Brunton, 2013). Furthermore, studies on the variation of the quality of maternal care in rats reported changes in dopaminergic responsivity to stress; adult rats which received low levels of maternal care displayed long lasting responses to stressors (Zhang et al., 2005; Ponchio et al., 2015). By contrast, it was recently reported that maternal separation had no detrimental effect on fear extinction memory (Zoicas and Neumann, 2016). Yet, although no elevation in anxiety-like or fear behaviors were observed for AB mice, one might assume that the efficacy to retain the newly formed memory for emotional learning was impaired due to maladaptive changes in overall neuronal plasticity during development.

Our data, assessed for mice of a mismatched, beneficial-adverse live history pairing (BA), points toward a higher state of arousal post fear conditioning, which declined over the course of extinction training. Although BA mice displayed reduced freezing during extinction training within the groups dynamics, freezing during R6 was still high in comparison to AB, BB, and sham-handled mice, indicating a delay in extinction learning. Interestingly, during extinction recall, these mice showed still a high percentage of freezing, on one hand, but reduced freezing behavior compared to AA und AB during extinction recall, indicating a distinct, life history–dependent modulation of emotional learning processes. Additionally, similar to AA life history, BA mice displayed initially increased levels of risk-assessment. Therefore, it can be assumed that chronic subordination with the possibility to escape indeed raises arousal in adult mice and disturbs emotional learning during training, which is in line with previous studies on social defeat (Korte and de Boer, 2003; Klinsey et al., 2007; Caldwell and Riccio, 2010; Hammels et al., 2015). Thus, future studies will be needed to identify the impact of this experience on fear memory, without possible counterbalancing factors of early life experiences.

Sham-handled animals, which served as a handling- and non-social control group, showed successful extinction and extinction recall. Compared to BB life history, overall behavioral expressions of these mice were similar, except quiet, which was not as prominent during extinction training. This finding might undermine our conclusion, that, though being handled, BB mice indeed displayed lower levels of stress-related behaviors after extinction training.

Next, assessment of “early vs. late phase” analysis showed that life history distinctly modulated individual behaviors. Chronic subordination during late life, negatively affected the emotional learning task by increasing levels of freezing and risk-assessment as it was observed in a number of previous studies (Korte and de Boer, 2003; Klinsey et al., 2007; Caldwell and Riccio, 2010; Hammels et al., 2015). Early life adversity negatively impacted freezing behavior during extinction recall, which is in line with Bingham et al. (2013). Specific combination of early and late phase shaped flight behavior and quiet in mice. An accumulation of adversity (AA) led to more flight responses, as an indicator of increased anxiety (Zhang et al., 2005; Ponchio et al., 2015), while a pairing of early and late benefit (BB) resulted in better adaption to cue presentation and experimental (environmental) condition. Taken together, these observations illustrate how the experience of positive and negative social-interactions during development impacts the overall behavioral outcome in an adult individual.

Freezing is considered to be a highly reliable behavioral readout when it comes to fear-related responses to adverse conditioned cues and is widely used to evaluate fear memory in rodents (Bouton and Bolles, 1979, 1980; Maren, 2008; Lesting et al., 2011b). Our findings on freezing duration to the first CS+ presentation of each session show that life history modifications and sham-handling did not affect fear memory retrieval. This suggests not only that each group successfully learned to associate the conditioned cue with an adverse event, but also the quality of fear memory was obtained to the same extent, thus no significant differences in freezing could be determined (Figure 7A). Furthermore, animals of each group could clearly differentiate between neutral and conditioned cue (Figure 6, Supplementary Figure S3) hereby excluding a possible effect of fear generalization due to prior treatment. Although previous studies reported that environmental enrichment improves cognitive function in mice (Doulames et al., 2014), and also stressing rats prenatally could already lead to fear generalization (Salm et al., 2015), no enhancing or reducing effects could be observed within this lifetime approach at the time point of fear retrieval.

Our results suggest that life history shaped fear extinction and consolidation for each life history setup in a distinct manner. Regarding a matched early and late life phase, represented by groups AA and BB, our data show that prior experience of early and late adversity (AA) is linked to an impairment of extinction learning and thus a disruption of its consolidation (Figure 6A). On the contrary, an early and late beneficial social environment (BB) seems to facilitate extinction learning, followed by internalization of the newly formed memory (Figures 6, 7). Several studies have reported that an accumulation of stressful events can lead to an impairment of fear extinction and consequently to diminished extinction memory consolidation in adult animals, which is in line with our findings in group AA (Baran et al., 2009; Green et al., 2011; Bingham et al., 2013). As discussed above, accumulation of beneficial socio-environmental experiences, as in BB life history, might contribute to lower vulnerability to stress in adult mice. However, opposing studies suggested that the absence of adversity is not inevitably associated with optimal outcomes (Seery et al., 2010, 2013). Sheltering from all stressors is assumed to prevent the individual to adequately develop coping strategies in their early life, while at least some adversity is considered to promote the ability to successfully cope with stressful events later on (Gluckman et al., 2007; Seery et al., 2010). Presentation of the conditioned stimulus creates a stressful (threatening) situation that is difficult to cope with or escape from. Therefore, a predictive adaptive response, as learned in a prior social context, is not immediately feasible.

Mice, which had faced a mismatch between early and late life were represented by groups AB and BA. Although, in this study, BA animals were capable to extinguish fear memory to some extent and to consolidate this newly formed memory, beneficial early life experiences could not fully compensate adverse life events during adulthood, especially when compared to other life history subjects in R6. High levels of arousal, indicated by an increase in risk-assessment even prior to the first neutral stimulus presentation, may have rendered them particularly vulnerable for further negative encounters in terms of the presented stimuli. One might speculate that elevated stress levels add to a corruption of underlying extinction mechanisms (Sachar et al., 1970; McEwen, 2003; Zhang et al., 2005; Bingham et al., 2013; Ponchio et al., 2015). In contrast, early life adversity followed by benefit during late phase (AB) enabled the subjects initially to perform extinction training and lowering basal arousal, but deteriorated extinction memory consolidation. In prior studies it was reported that at least some adversity in an individuals' early life would function as a precursor for resilience to further stressors encountered in late life (Seery et al., 2013). Additionally, it was also shown in rats that mating success in late life was correlated to lower anxiety and elevated levels of oxytocin which could also function as a shelter against upcoming adverse events (Waldherr and Neumann, 2007). Nevertheless, it can be assumed that a mismatch in life history experiences (BA and AB) mainly influenced extinction memory consolidation.

We show that adverse early life events (AA and AB) as compared to early life benefit (BA and BB) impacted consolidation of fear extinction memory, whereas late life adversity (AA and BA) affected extinction learning. To our knowledge, there is no study investigating the impact of adverse early life events on extinction consolidation as used in our paradigm. However, studies in rats revealed that early life adversity can induce depressive-like behavior, deficits in social behavior and modulation of emotional learning processes later in life (Moriceau et al., 2009; Callaghan and Richardson, 2012; Raineki et al., 2012). One might speculate that the experience of early live adversity leads to morphological changes in still developing brain structures, needed for fear learning and extinction processes. It was reported that adversity in early life, such as maternal separation, indeed reduced neurogenesis and generated morphological changes in the hippocampus and medial prefrontal cortex (Lajud et al., 2012; Soztutar et al., 2016). Together with the amygdala, these brain structures form a tripartite system which governs fear learning, extinction processes and memory consolidation (LeDoux, 2000; Milad and Quirk, 2002; Maren and Quirk, 2004; Likhtik et al., 2005; Quirk et al., 2006). Lesion studies in rats showed that especially the prefrontal cortex contributed to extinction memory consolidation (Quirk et al., 2000; Zelinski et al., 2010).

There is no data on extinction learning in mice which experienced chronic subordination with a possibility to escape. Thus, there are some indicators for chronic stressors, such as repeated loser experiences in adult animals (Korte and de Boer, 2003; Klinsey et al., 2007; Caldwell and Riccio, 2010; Hammels et al., 2015). Social defeat was reported to increase anxiety-like behavior, but did not affect extinction learning in 5-HTT mice (Narayanan et al., 2011). Interestingly, loser experience in heterozygous 5-HTT mice, which initially behaved like 5-HTT mice, resulted in an impairment of fear extinction. It can be speculated that a further modulator, in this case the genetic pre-disposition, or an adverse early life environment as used in the present study, might lead to a shift toward an impairment of fear extinction as seen in AA life history. Hence, BA animals showed no impairment of fear extinction, although freezing was significantly increased as compared to BB and AB in R6. Finally, as discussed above, our life history approach, comprising four stages of life (prenatal and early postnatal phase, adolescence, and adulthood), was assigned to two experimental phases, i.e., early phase and late phase. Thereby, three out of the four life stages (prenatal phase, early postnatal phase, adolescence) were combined to create either “beneficial” or “adverse” early life conditions, while “beneficial” or “adverse” late life conditions involved experiences exclusively made during adulthood. The rather long period of early life experiences was thus contrasted with a short intervention during adulthood. The adversity experienced in the early phase lasted thus much longer than the beneficial conditions experienced in later life. A short beneficial experience in adulthood may therefore be too subtle to buffer or even reverse the chronic character of adverse experiences made in early phases of life, and might point to a putative timely imbalance between the defined early and late life phase. Yet, this study was dedicated to mimic a match/mismatch condition in analogy to human life histories to gain a deeper understanding on the overall impact of whole life experiences. Follow up studies will build on this gained knowledge and focus specifically on distinct life phases.

## Conclusion

Our study shows that life history explicitly shaped anxiety-like behavior as well as fear memory and extinction. Notably, anxiety-like behavior and fear extinction were differently expressed depending on the life history background. The distinction between anxiety and fear becomes particularly evident as a beneficial early and adverse later mismatched life history (BA) positively impacted anxiety-like behavior, whereas such life experience led to a disruption of extinction memory consolidation. Correspondingly, accumulation of beneficial experiences throughout lifetime (BB) led to higher levels of anxiety-like behavior, but facilitated extinction learning and extinction memory consolidation. Finally, accumulation of adverse life experiences (AA) led to an impairment of fear extinction and disruption of extinction memory consolidation as compared to a throughout beneficial life history (BB). In contrast, anxiety-like behavior did not differ significantly between these two life history groups.

Concerning anxiety-like behavior, the results do neither support the allostatic load nor the mismatch hypothesis, but rather indicate an anxiolytic effect of a mismatched early beneficial and later adverse life history. In contrast, fear memory was strongly affected by the accumulation of adverse experiences over the lifetime, supporting the allostatic load hypothesis. In summary, anxiety-like behavior and fear memory are differentially shaped during lifetime development. These results stress the need for comprehensive information on molecular and neuronal processes of anxiety- and fear-related brain structures throughout an individual's life history.

## Author contributions

Substantial contributions to the conception or design of the work (NS, H-CP, TS); or the acquisition (JR, CB), analysis (JR, CB, JL), or interpretation of data for the work (JR, CB); and drafting the work (JR, CB, TS) or revising it critically for important intellectual content (JR, CB, SHR, JL, NS, H-CP, TS); and final approval of the version to be published (JR, CB, SHR, JL, NS, H-CP, TS); and agreement to be accountable for all aspects of the work in ensuring that questions related to the accuracy or integrity of any part of the work are appropriately investigated and resolved (JR, CB, SHR, JL, NS, H-CP, TS).

## Funding

This work was supported by the German Research Foundation (DFG, SFB-TRR58, TP A01 to NS and TS and TP A02 to TS and H-CP).

### Conflict of interest statement

The authors declare that the research was conducted in the absence of any commercial or financial relationships that could be construed as a potential conflict of interest.

## Supplementary material

Supplementary Figure S1 **Influence of life history on body weights**. Body weights were assessed at weaning (PND 22) after simulation of either dangerous or safe environment, at the end of the early phase (PND 70 ± 2) after 5 loser or mating experiences, and at the end of the late phase (PND 77 ± 2) after escapable defeat or cohabitation with a female. Data are presented as means ±SEM. Statistics: ANOVA; *post-hoc* testing: ANOVA with repeated measures. ^*^*p* < 0.05; ^***^*p* < 0.001. Animals per group: AA = 28, AB = 27, BA = 28, BB = 27, SH = 28.

Supplementary Figure S2 **Influence of life history on behavioral expressions after conditioned fear. (A)** Fear retrieval (R1): Proportional values of displayed behaviors show a general increase of anxiety-related behaviors (freezing, risk-assessment and flight) upon CS presentations in all groups during R1 session. Non-adverse, active behaviors, such as grooming, exploration and rearing, were mostly observed prior, between and post stimulus presentations. Animals of AA and BA life history showed proportionally elevated levels of risk-assessment prior to the first stimulus presentation. **(B)** Fear extinction: Defensive responses became less during final session of extinction learning (R6). AA and BA mice still displayed elevated defensive behaviors during stimuli presentations. BB animals were mostly quiet. **(C)** Extinction recall (E): Defensive responses were mainly observed in response to stimuli presentations, whereas AA and AB mice showed a strong augmentation during CS+ presentation in comparison to other groups. Color code of different behaviors is illustrated; animals per group: *n* = 15.

Supplementary Figure S3 **Expression of freezing (% of time) in response to first CS− presentation during retrieval (R1), extinction (R6), and extinction recall (E) dependent on different life histories. (A)** Animals of all life history groups and **(B)** sham-handled mice significantly reduced their freezing response to the neutral stimulus (R1 vs. R6 and E). Furthermore, comparison to freezing levels in response to the first CS+ revealed, that all groups were able to distinguish between the stimuli. Therefore, no generalization effect could be detected. ^***^*p* < 0.001; animals per group: *n* = 15.

Supplementary Table S1 **Influence of life history on discrete behavioral expressions after fear conditioning (% of time)**. Life histories: AA, early and late adversity; AB, early adversity and late benefit; BA, early benefit and late adversity; BB, early and late benefit; SH, sham-handled. Fear memory states: R1, retrieval session 1; R6, retrieval session 6 (end of extinction learning); E, extinction recall. Discrete behavioral expressions (freezing, risk-assessment, flight, grooming, exploration, rearing, quiet; % of time) was scored before, during and after CS− and CS+ presentation, respectively. Animals per group: *n* = 15.

## Appendix

### Body weight

Over the course of each life history (PND 22, 70 ± 2, and 77 ± 2) body weights were measured.

Body weights changed over the course of life, demonstrated by a significant main effect of time [Huynh-Feldt correction: *F*_(1.7, 226.6)_ = 27572.696, *p* < 0.001; Supplementary Figure S1]. Bonferroni *post-hoc* analysis revealed a significant weight increase from PND 22 to 70 ± 2 (8.78 ± 0.09 vs. 23.69 ± 0.12; *p* < 0.001) as well as from PND 70 ± 2 to 77 ± 2 (23.69 ± 0.12 vs. 24.20 ± 0.11; *p* < 0.001).

Furthermore, a significant main effect of life history was detected [*F*_(4, 133)_ = 3.373, *p* < 0.05] as well as a significant life history-by-time interaction [Huynh-Feldt correction: *F*_(6.8, 226.6)_ = 4.813, *p* < 0.001], with body weights increasing from PND 22 to PND 70 ± 2 in mice of each life history (*p* < 0.001). From PND 70 ± 2 to 77 ± 2, a significant increase in weights was found in mice of four life history groups (AA: *p* < 0.001; BA: *p* < 0.001; BB: *p* < 0.001; SH: *p* < 0.05).

The analysis of body weights at the three different time points revealed significant main effects of life history on weights at PND 22 [*F*_(4, 133)_ = 3.067, *p* < 0.05], PND 70 ± 2 [*F*_(4, 133)_ = 5.033, *p* < 0.001]. *Post-hoc* analysis showed that on PND 22, AA mice weighed significantly more than SH mice (*p* < 0.05). On PND 70 ± 2, AB mice had significantly higher body weights compared to BA (*p* < 0.01) and BB mice (*p* < 0.01).

To disentangle specific effects of the early and the late phase, an additional “early vs. late phase” analysis was conducted. A significant main effect of the early phase on body weights at PND 22 [*F*_(1, 106)_ = 4.186, *p* < 0.05], PND 70 ± 2 [*F*_(1, 106)_ = 17.691, *p* < 0.001] and PND 77 ± 2 [*F*_(1, 106)_ = 7.435, *p* < 0.01] was detected, with mice that experienced early adversity (AA and AB) having higher body weights at both sampling points compared to mice of the early beneficial condition (BA and BB). No significant main effects of the late phase were found.

In brief summary, animals of all groups increased their body weight throughout development. At PND 22, AA animals showed highest body weight as compared to SH. At PND 70 ± 2, AB mice weighed more than BA and BB, while no significant differences could be detected at PND 77 ± 2. Experience of early life adversity (AA and AB) led to higher body weights as in contrast to early life benefit (BA and BB) at PND 70 ± 2 and 77 ± 2.

### Behavioral expressions after conditioned fear (exploration, quiescence, grooming rearing; supplementary figure S2 and supplementary table S1)

#### Exploration

Exploration was observed during all sessions. Analysis revealed a significant interaction effect of time period × session × life history interaction on explorative behavior [*F*_(32, 560)_ = 2.201, *p* < 0.001]. *Post-hoc* analysis demonstrated that AA mice showed a significant diminishment in exploratory movements from R1 to R6 during preCS− (*p* < 0.001) and postCS+ (*p* < 0.001). Additionally, exploration duration significantly increased from R6 to E during CS− presentation (*p* < 0.05). BB mice significantly reduced exploratory movements during extinction training in postCS+ (*p* < 0.001), while exploration was significantly decreased for preCS+ at R6 compared to R1 (*p* < 0.001) and E (*p* < 0.05). Animals of a mismatched life history (BA and AB) and sham-handled mice showed significantly diminished exploration duration during postCS+ at R6 (*p* < 0.001), while BA mice displayed a significant increase from R6 to E (*p* < 0.001). Furthermore, a significant increase of exploratory movements could be observed during CS− presentation for SH mice (*p* < 0.001) at the end of extinction training. Early and late phase effects on exploration, utilizing “early vs. late phase” analysis, excluding SH animals, detected no interaction effect.

#### Quiescence

Quiescence was mainly observed during the final extinction session R6. Analysis revealed a significant interaction effect of time period × session × life history [*F*_(32, 560)_ = 1.845, *p* < 0.01] on quiet. *Post-hoc* analysis regarding AA group showed that time spent quiet was significantly increased for preCS− during R6 and E compared to R1 (*p* < 0.001). Also, a significant increase could be detected for preCS+ and postCS+ at R6 (*p* < 0.001) and for postCS+ at E as compared to R1 (*p* < 0.01). Animals of BB group were significantly quiet in all time periods of session R6 as compared to R1 (*p* < 0.001) and E (*p* < 0.001). Furthermore, quiet was also significantly decreased during postCS+ at R6 when compared to E (*p* < 0.05). Animals of mismatched life histories were most quiet at R6, with BA mice displaying this behavior during all time periods compared to R1 (preCS−, preCS+, CS+, and postCS+: *p* < 0.001; CS−: *p* < 0.01) and additionally during postCS+ in E (*p* < 0.001). AB mice showed the following diverging pattern: during preCS−, time spent quiet at R6 was significantly elevated compared to E (*p* < 0.01) and not to R1. Except during preCS+, a significant effect was detected for time periods CS− (*p* < 0.01), CS+ and postCS+ (*p* < 0.001) at R6 as compared to R1. At E significant effects were detected for CS+ and postCS+ as in comparison to R1 (*p* < 0.001).

Notably, between-group comparisons revealed that BB animals spent significantly more time quiet during CS− presentation in contrast to AA (*p* < 0.01), BA (*p* < 0.01), and AB (*p* < 0.05), while during CS+ this effect was found only for comparison of AA and BB mice (*p* < 0.01).

Early and late phase effects on quiet, detected an interaction effect of session × time period × early phase × late phase [*F*_(8, 448)_ = 2.711, *p* < 0.01]. *Post-hoc* analysis demonstrated that combination of early and late benefit compared to animals which experienced adversity in any life phase, resulted in a significant increase in time spent quiet during CS− presentation at R6 (AA and BA, *p* < 0.01; AB, *p* < 0.05). This effect was also observed during CS+ and postCS+ time period at R6, if early and late adversity was compared to early and late benefit (CS+, *p* < 0.01; postCS+, *p* < 0.05).

#### Grooming and rearing

Analysis of grooming and rearing behavior revealed no significant influence of life history.

## References

[B1] Archer J. (1973). Tests for emotionality in rats and mice - review. Anim. Behav. 21, 205–235. 10.1016/S0003-3472(73)80065-X 4578750

[B2] Baran S. E. Armstrong C. E. Niren D. C. Hanna J. J. Conrad C. D. (2009). Chronic stress and sex differences on the recall of fear conditioning and extinction. Neurobiol. Learn. Mem. 91, 321–330. 10.1016/j.nlm.2008.11.005 19073269 PMC2673234

[B3] Bartolomucci A. Pederzani T. Sacerdote P. Panerai A. E. Parmigiani S. Palanza P. (2004). Behavioral and physiological characterization of male mice under chronic psychosocial stress. Psychoneuroendocrinology 29, 899–910. 10.1016/j.psyneuen.2003.08.003 15177705

[B4] Bateson P. Barker D. Clutton-Brock T. Deb D. D'Udine B. Foley R. A.. (2004). Developmental plasticity and human health. Nature 430, 419–421. 10.1038/nature02725 15269759

[B5] Bateson P. Gluckman P. Hanson M. (2014). The biology of developmental plasticity and the Predictive Adaptive Response hypothesis. J. Physiol. 592, 2357–2368. 10.1113/jphysiol.2014.271460 24882817 PMC4048093

[B6] Belsky J. Pluess M. (2009). Beyond diathesis stress: differential susceptibility to environmental influences. Psychol. Bull. 135, 885–908. 10.1037/a0017376 19883141

[B7] Bingham B. C. Rani C. S. S. Frazer A. Strong R. Morilak D. A. (2013). Exogenous prenatal corticosterone exposure mimics the effects of prenatal stress on adult brain stress response systems and fear extinction behavior. Psychoneuroendocrinology 38, 2746–2757. 10.1016/j.psyneuen.2013.07.003 23937971

[B8] Blanchard D. C. Griebe L. G. Blanchard R. J. (2003). The Mouse Defense Test Battery: pharmacological and behavioral assays for anxiety and panic. Eur. J. Pharmacol. 463, 197–116. 10.1016/S0014-2999(03)01276-7 12600704

[B9] Blanchard R. J. Griebel G. Henrie J. A. Blanchard D. C. (1997). Differentiation of anxiolytic and panicolytic drugs by effects on rat and mouse defense test batteries. Neurosci. Biobehav. Rev. 21, 783–789. 10.1016/S0149-7634(96)00062-0 9415903

[B10] Bodden C. Richter S. H. Schreiber R. S. Kloke V. Gerss J. Palme R.. (2015). Benefits of adversity?! How life history affects the behavioral profile of mice varying in serotonin transporter genotype. Front. Behav. Neurosci. 9:47. 10.3389/fnbeh.2015.00047 25784864 PMC4347490

[B11] Bouton M. E. Bolles R. C. (1979). Role of conditioned contextual stimuli in reinstatement of extinguished fear. J. Exp. Psychol. Anim. Behav. Process 5, 368–378. 10.1037/0097-7403.5.4.368 528893

[B12] Bouton M. E. Bolles R. C. (1980). Conditioned fear assessed by freezing and by the suppression of three different baselines. Anim. Learn. Behav. 8, 429–434. 10.3758/BF03199629

[B13] Brunton P. J. (2013). Effects of maternal exposure to social stress during pregnancy: consequences for mother and offspring. Reproduction 146, R175–R189. 10.1530/rep-13-0258 23901130

[B14] Buwalda B. Kole M. H. P. Veenema A. H. Huininga M. de Boer S. F. Korte S. M.. (2005). Long-term effects of social stress on brain and behavior: a focus on hippocampal functioning. Neurosci. Biobehav. Rev. 29, 83–97. 10.1016/j.neubiorev.2004.05.005 15652257

[B15] Caldji C. Tannenbaum B. Sharma S. Francis D. Plotsky P. M. Meaney M. J. (1998). Maternal care during infancy regulates the development of neural systems mediating the expression of fearfulness in the rat. Proc. Natl. Acad. Sci. U.S.A. 95, 5335–5340. 10.1073/pnas.95.9.5335 9560276 PMC20261

[B16] Caldwell E. E. Riccio D. C. (2010). Alcohol self-administration in rats: modulation by temporal parameters related to repeated mild social defeat stress. Alcohol 44, 265–274. 10.1016/j.alcohol.2010.02.012 20682194

[B17] Calhoon G. G. Tye K. M. (2015). Resolving the neural circuits of anxiety. Nat. Neurosci. 18, 1394–1404. 10.1038/nn.4101 26404714 PMC7575249

[B18] Callaghan B. L. Richardson R. (2012). The effect of adverse rearing environments on persistent memories in young rats: removing the brakes on infant fear memories. Transl. Psychiatry 2:e138. 10.1038/tp.2012.65 22781171 PMC3410617

[B19] Chaby L. E. Cavigelli S. A. Hirrlinger A. M. Caruso M. J. Braithwaite V. A. (2015). Chronic unpredictable stress during adolescence causes long-term anxiety. Behav. Brain Res. 278, 492–495. 10.1016/j.bbr.2014.09.003 25448433

[B20] Cratty M. S. Ward H. E. Johnson E. A. Azzaro A. J. Birkle D. L. (1995). Prenatal stress increases corticotropin-releasing factor (CRF) content and release in rat amygdala minces. Brain Res. 675, 297–302. 10.1016/0006-8993(95)00087-7 7796142

[B21] Crawley J. Goodwin F. K. (1980). Preliminary-report of a simple animal behavior model for the anxiolytic effects of benzodiazepines. Pharmacol. Biochem. Behav. 13, 167–170. 10.1016/0091-3057(80)90067-2 6106204

[B22] Dantzer B. Newman A. E. Boonstra R. Palme R. Boutin S. Humphries M. M.. (2013). Density triggers maternal hormones that increase adaptive offspring growth in a wild mammal. Science 340, 1215–1217. 10.1126/science.1235765 23599265

[B23] de Kloet E. R. Joels M. Holsboer F. (2005). Stress and the brain: from adaptation to disease. Nat. Rev. Neurosci. 6, 463–475. 10.1038/nrn1683 15891777

[B24] Dienstbier R. A. (1989). Arousal and physiological toughness: implications for mental and physical health. Psychol. Rev. 96, 84–100. 10.1037/0033-295X.96.1.84 2538855

[B25] Doulames V. Lee S. Shea T. B. (2014). Environmental enrichment and social interaction improve cognitive function and decrease reactive oxidative species in normal adult mice. Int. J. Neurosci. 124, 369–376. 10.3109/00207454.2013.848441 24102158

[B26] Edinger K. L. Frye C. A. (2007). Sexual experience of male rats influences anxiety-like behavior and androgen levels. Physiol. Behav. 92, 443–453. 10.1016/j.physbeh.2007.04.018 17544460

[B27] Eiland L. Ramroop J. Hill M. N. Manley J. McEwen B. S. (2012). Chronic juvenile stress produces corticolimbic dendritic architectural remodeling and modulates emotional behavior in male and female rats. Psychoneuroendocrinology 37, 39–47. 10.1016/j.psyneuen.2011.04.015 21658845 PMC3181388

[B28] Fanselow M. S. Bolles R. C. (1979). Naloxone and shock-elicited freezing in the rat. J. Comp. Physiol. Psychol. 93, 736–744. 10.1037/h0077609 479405

[B29] Fontana A. Rosenheck R. (1998). Psychological benefits and liabilities of traumatic exposure in the war zone. J. Trauma. Stress 11, 485–503. 10.1023/A:1024452612412 9690189

[B30] Gerlai R. (1998). Contextual learning and cue association in fear conditioning in mice: a strain comparison and a lesion study. Behav. Brain Res. 95, 191–203. 10.1016/S0166-4328(97)00144-7 9806439

[B31] Gluckman P. D. Hanson M. A. Beedle A. S. (2007). Early life events and their consequences for later disease: a life history and evolutionary perspective. Am. J. Hum. Biol. 19, 1–19. 10.1002/ajhb.20590 17160980

[B32] Gluckman P. D. Hanson M. A. Spence R. H. G. Bateson P. (2005b). Environmental influences during development and their later consequences for health and disease: implications for the interpretation of empirical studies. Proc. R. Soc. Biol. Sci. 272, 671–677. 10.1098/rspb.2004.3001 15870029 PMC1602053

[B33] Gluckman P. D. Hanson M. A. Morton S. M. B. Pinal C. S. (2005a). Life-long echoes - A critical analysis of the developmental origins of adult disease model. Biol. Neonate 87, 127–139. 10.1159/000082311 15564779

[B34] Green M. K. Rani C. S. S. Joshi A. Soto-Pina A. E. Martinez P. A. Frazer A.. (2011). Prenatal stress induces long term stress vulnerability, compromising stress response systems in the brain and impairing extinction of conditioned fear after adult stress. Neuroscience 192, 438–451. 10.1016/j.neuroscience.2011.06.041 21723377

[B35] Gross C. Hen R. (2004). The developmental origins of anxiety. Nat. Rev. Neurosci. 5, 545–552. 10.1038/nrn1429 15208696

[B36] Grossen N. E. Kelley M. J. (1972). Species-specific behavior and acquisition of avoidance behavior in rats. J. Comp. Physiol. Psychol. 81, 307–310. 10.1037/h0033536 5084446

[B37] Hammels C. Pishva E. De V. J. Van den Hove D. L. Prickaerts J. Van W. R.. (2015). Defeat stress in rodents: from behavior to molecules. Neurosci. Biobehav. Rev. 59, 111–140. 10.1016/j.neubiorev.2015.10.006 26475995

[B38] Heiming R. S. Bodden C. Jansen F. Lewejohann L. Kaiser S. Lesch K. P.. (2011). Living in a dangerous world decreases maternal care: a study in serotonin transporter knockout mice. Horm. Behav. 60, 397–407. 10.1016/j.yhbeh.2011.07.006 21787775

[B39] Heiming R. S. Jansen F. Lewejohann L. Kaiser S. Schmitt A. Lesch K. P.. (2009). Living in a dangerous world: the shaping of behavioral profile by early environment and 5-HTT genotype. Front. Behav. Neurosci. 3:26. 10.3389/neuro.08.026.2009 19826611 PMC2759357

[B40] Hubel D. H. Wiesel T. N. (1970). The period of susceptibility the physiological effects of unilateral eye closure in kittens. J. Physiol. 206, 419–436. 10.1113/jphysiol.1970.sp009022 5498493 PMC1348655

[B41] Jansen F. Heiming R. S. Lewejohann L. Touma C. Palme R. Schmitt A.. (2010). Modulation of behavioural profile and stress response by 5-HTT genotype and social experience in adulthood. Behav. Brain Res. 207, 21–29. 10.1016/j.bbr.2009.09.033 19782704

[B42] Kaiser S. Sachser N. (2005). The effects of prenatal social stress on behaviour: mechanisms and function. Neurosci. Biobehav. Rev. 29, 283–294. 10.1016/j.neubiorev.2004.09.015 15811499

[B43] Kästner N. Richter S. H. Lesch K. P. Schreiber R. S. Kaiser S. Sachser N. (2015). Benefits of a “vulnerability gene”? A study in serotonin transporter knockout mice. Behav. Brain Res. 283, 116–120. 10.1016/j.bbr.2015.01.031 25629942

[B44] Klinsey S. G. Bailey M. T. Sheridan J. F. Padgett D. A. Avitsur R. (2007). Repeated social defeat causes increased anxiety-like behavior and alters splenocyte function in C57BL/6 and CD-1 mice. Brain Behav. Immun. 21, 458–466. 10.1016/j.bbi.2006.11.001 17178210 PMC1941837

[B45] Kloke V. Jansen F. Heiming R. S. Palme R. Lesch K. P. Sachser N. (2011). The winner and loser effect, serotonin transporter genotype, and the display of offensive aggression. Physiol. Behav. 103, 565–574. 10.1016/j.physbeh.2011.04.021 21549735

[B46] Korte S. M. de Boer S. F. (2003). A robust animal model of state anxiety: fear-potentiated behaviour in the elevated plus-maze. Eur. J. Pharmacol. 463, 163–175. 10.1016/S0014-2999(03)01279-2 12600708

[B47] Lajud N. Roque A. Cajero M. Gutierrez-Ospina G. Torner L. (2012). Periodic maternal separation decreases hippocampal neurogenesis without affecting basal corticosterone during the stress hyporesponsive period, but alters HPA axis and coping behavior in adulthood. Psychoneuroendocrinology 37, 410–420. 10.1016/j.psyneuen.2011.07.011 21862224

[B48] Laxmi T. R. Stork O. Pape H. C. (2003). Generalisation of conditioned fear and its behavioural expression in mice. Behav. Brain Res. 145, 89–98. 10.1016/S0166-4328(03)00101-3 14529808

[B49] LeDoux J. E. (1993). Emotional memory: in search of systems and synapses. Ann. N.Y. Acad. Sci. 702, 149–157. 10.1111/j.1749-6632.1993.tb17246.x 8109874

[B50] LeDoux J. E. (2000). Emotion circuits in the brain. Ann. Rev. Neurosci. 23, 155–184. 10.1146/annurev.neuro.23.1.155 10845062

[B51] Lee E. J. Son G. H. Chung S. Lee S. Kim J. Choi S.. (2011). Impairment of fear memory consolidation in maternally stressed male mouse offspring: evidence for nongenomic glucocorticoid action on the amygdala. J. Neurosci. 31, 7131–7140. 10.1523/JNEUROSCI.4692-10.2011 21562275 PMC6703196

[B52] Lesting J. Daldrup T. Narayanan V. Himpe C. Seidenbecher T. Pape H. C. (2013). Directional theta coherence in prefrontal cortical to amygdalo-hippocampal pathways signals fear extinction. PLoS ONE 8:e77707. 10.1371/journal.pone.0077707 24204927 PMC3812006

[B53] Lesting J. Geiger M. Narayanan R. T. Pape H. C. Seidenbecher T. (2011a). Impaired extinction of fear and maintained amygdala-hippocampal theta synchrony in a mouse model of temporal lobe epilepsy. Epilepsia 52, 337–346. 10.1111/j.1528-1167.2010.02758.x 21054349

[B54] Lesting J. Narayanan R. T. Kluge C. Sangha S. Seidenbecher T. Pape H. C. (2011b). Patterns of coupled theta activity in amygdala-hippocampal-prefrontal cortical circuits during fear extinction. PLoS ONE 6:e21714. 10.1371/journal.pone.0021714 21738775 PMC3125298

[B55] Lewejohann L. Kloke V. Heiming R. S. Jansen F. Kaiser S. Schmitt A.. (2010). Social status and day-to-day behaviour of male serotonin transporter knockout mice. Behav. Brain Res. 211, 220–228. 10.1016/j.bbr.2010.03.035 20347882

[B56] Likhtik E. Pelletier J. G. Paz R. Pare D. (2005). Prefrontal control of the amygdala. J. Neurosci. 25, 7429–7437. 10.1523/JNEUROSCI.2314-05.2005 16093394 PMC6725290

[B57] Lister R. G. (1987). The use of a plus-maze to measure anxiety in the mouse. Psychopharmacology 92, 180–185. 10.1007/BF00177912 3110839

[B58] Lister R. G. (1990). Ethologically-based animal-models of anxiety disorders. Pharmacol. Therapeutics 46, 321–340. 10.1016/0163-7258(90)90021-S 2188266

[B59] Maren S. (2008). Pavlovian fear conditioning as a behavioral assay for hippocampus and amygdala function: cautions and caveats. Eur. J. Neurosci. 28, 1661–1666. 10.1111/j.1460-9568.2008.06485.x 18973583

[B60] Maren S. Quirk G. J. (2004). Neuronal signalling of fear memory. Nat. Rev. Neurosci. 5, 844–852. 10.1038/nrn1535 15496862

[B61] McCormick C. M. Smith C. Mathews I. Z. (2008). Effects of chronic social stress in adolescence on anxiety and neuroendocrine response to mild stress in male and female rats. Behav. Brain Res. 187, 228–238. 10.1016/j.bbr.2007.09.005 17945360

[B62] McEwen B. S. (2003). Mood disorders and allostatic load. Biol. Psychiatry 54, 200–207. 10.1016/S0006-3223(03)00177-X 12893096

[B63] McEwen B. S. (2006). Protective and damaging effects of stress mediators: central role of the brain. Dial. Clin. Neurosci. 8, 367–381. 17290796 10.31887/DCNS.2006.8.4/bmcewen PMC3181832

[B64] Meaney M. J. (2001). Maternal care, gene expression, and the transmission of individual differences in stress reactivity across generations. Annu. Rev. Neurosci. 24, 1161–1192. 10.1146/annurev.neuro.24.1.1161 11520931

[B65] Meyer N. Richter S. H. Schreiber R. S. Kloke V. Kaiser S. Lesch K. P.. (2016). The Unexpected effects of beneficial and adverse social experiences during adolescence on anxiety and aggression and their modulation by genotype. Front. Behav. Neurosci. 10:97. 10.3389/fnbeh.2016.00097 27303275 PMC4880570

[B66] Milad M. R. Quirk G. J. (2002). Neurons in medial prefrontal cortex signal memory for fear extinction. Nature 420, 70–74. 10.1038/nature01138 12422216

[B67] Moriceau S. Raineki C. Holman J. D. Holman J. G. Sullivan R. M. (2009). Enduring neurobehavioral effects of early life trauma mediated through learning and corticosterone suppression. Front. Behav. Neurosci. 3:22. 10.3389/neuro.08.022.2009 19750195 PMC2741290

[B68] Myers K. M. Davis M. (2007). Mechanisms of fear extinction. Mol. Psychiatry 12, 120–150. 10.1038/sj.mp.4001939 17160066

[B69] Narayanan V. Heiming R. S. Jansen F. Lesting J. Sachser N. Pape H. C.. (2011). Social defeat: impact on fear extinction and amygdala-prefrontal cortical theta synchrony in 5-HTT deficient mice. PLoS ONE 6:e22600. 10.1371/journal.pone.0022600 21818344 PMC3144906

[B70] Navarro J. F. (1997). An ethoexperimental analysis of the agonistic interactions in isolated male mice: comparison between OF. 1 and NMRI strains. Psicothema 9, 333–336.

[B71] Nestler E. J. (2012). Epigenetics: stress makes its molecular mark. Nature 490, 171–172. 10.1038/490171a 23060173 PMC4858713

[B72] Pape H. C. Pare D. (2010). Plastic synaptic networks of the amygdala for the acquisition, expression, and extinction of conditioned fear. Physiol. Rev. 90, 419–463. 10.1152/physrev.00037.2009 20393190 PMC2856122

[B73] Parker K. J. Buckmaster C. L. Schatzberg A. F. Lyons D. M. (2004). Prospective investigation of stress inoculation in young monkeys. Arch. Gen. Psychiatry 61, 933–941. 10.1001/archpsyc.61.9.933 15351772

[B74] Parker K. J. Buckmaster C. L. Sundlass K. Schatzberg A. F. Lyons D. M. (2006). Maternal mediation, stress inoculation, and the development of neuroendocrine stress resistance in primates. Proc. Natl. Acad. Sci. U.S.A. 103, 3000–3005. 10.1073/pnas.0506571103 16473950 PMC1413772

[B75] Pellow S. Chopin P. File S. E. Briley M. (1985). Validation of open - closed arm entries in an elevated plus-maze as a measure of anxiety in the rat. J. Neurosci. Meth. 14, 149–167. 10.1016/0165-0270(85)90031-7 2864480

[B76] Pohl J. Olmstead M. C. Wynne-Edwards K. E. Harkness K. Menard J. L. (2007). Repeated exposure to stress across the childhood-adolescent period alters rats' anxiety- and depression-like behaviors in adulthood: the importance of stressor type and gender. Behav. Neurosci. 121, 462–474. 10.1037/0735-7044.121.3.462 17592937

[B77] Ponchio R. A. Teodorov E. Kirsten T. B. Coelho C. P. Oshiro A. Florio J. C.. (2015). Repeated methylphenidate administration during lactation reduces maternal behavior, induces maternal tolerance, and increases anxiety-like behavior in pups in adulthood. Neurotoxicol. Teratol. 50, 64–72. 10.1016/j.ntt.2015.05.008 26022000

[B78] Quirk G. J. Beer J. S. (2006). Prefrontal involvement in the regulation of emotion: convergence of rat and human studies. Curr. Opin. Neurobiol. 16, 723–727. 10.1016/j.conb.2006.07.004 17084617

[B79] Quirk G. J. Garcia R. Gonzalez-Lima F. (2006). Prefrontal mechanisms in extinction of conditioned fear. Biol. Psychiatry 60, 337–343. 10.1016/j.biopsych.2006.03.010 16712801

[B80] Quirk G. J. Russo G. K. Barron J. L. Lebron K. (2000). The role of ventromedial prefrontal cortex in the recovery of extinguished fear. J. Neurosci. 20, 6225–6231. 10934272 10.1523/JNEUROSCI.20-16-06225.2000 PMC6772571

[B81] Raineki C. Cortés M. R. Belnoue L. Sullivan R. M. (2012). Effects of early-life abuse differ across development: infant social behavior deficits are followed by adolescent depressive-like behaviors mediated by the amygdala. J. Neurosci. 32, 7758–7765. 10.1523/JNEUROSCI.5843-11.2012 22649253 PMC3488459

[B82] Ricon T. Toth E. Leshem M. Braun K. Richter-Levin G. (2012). Unpredictable chronic stress in juvenile or adult rats has opposite effects, respectively, promoting and impairing resilience. Stress 15, 11–20. 10.3109/10253890.2011.572207 21682654

[B83] Sachar E. J. Hellman L. Fukushima D. K. Gallaghe T. F. (1970). Cortisol production in depressive illness - a clinical and biochemical clarification. Arch. Gen. Psychiatry 23, 289–298. 10.1001/archpsyc.1970.01750040001001 4918519

[B84] Sachser N. Hennessy M. B. Kaiser S. (2011). Adaptive modulation of behavioural profiles by social stress during early phases of life and adolescence. Neurosci. Biobehav. Rev. 35, 1518–1533. 10.1016/j.neubiorev.2010.09.002 20854842

[B85] Sachser N. Kaiser S. Hennessy M. B. (2013). Behavioural profiles are shaped by social experience: when, how and why. Philos. Trans. R. Soc. B. 368:20120344. 10.1098/rstb.2012.0344 23569292 PMC3638447

[B86] Salm A. K. Lally B. E. Borysiewicz E. Fil D. Konat G. (2015). Analysis of extinction acquisition to attenuated tones in prenatally stressed and non-stressed offspring following auditory fear conditioning. Physiol. Behav. 139, 157–166. 10.1016/j.physbeh.2014.11.027 25449394

[B87] Sangha S. Narayanan R. T. Bergado-Acosta J. R. Stork O. Seidenbecher T. Pape H. C. (2009). Deficiency of the 65 kDa isoform of glutamic acid decarboxylase impairs extinction of cued but not contextual fear memory. J. Neurosci. 29, 15713–15720. 10.1523/JNEUROSCI.2620-09.2009 20016086 PMC6666166

[B88] Santarelli S. Lesuis S. L. Wang X. D. Wagner K. V. Hartmann J. Labermaier C.. (2014). Evidence supporting the match/mismatch hypothesis of psychiatric disorders. Eur. Neuropsychopharmacol. 24, 907–918. 10.1016/j.euroneuro.2014.02.002 24589292

[B89] Schmidt M. V. (2011). Animal models for depression and the mismatch hypothesis of disease. Psychoneuroendocrinology 36, 330–338. 10.1016/j.psyneuen.2010.07.001 20674180

[B90] Schmidt M. V. Sterlemann V. Ganea K. Liebl C. Alam S. Harbich D.. (2007). Persistent neuroendocrine and behavioral effects of a novel, etiologically relevant mouse paradigm for chronic social stress during adolescence. Psychoneuroendocrinology 32, 417–429. 10.1016/j.psyneuen.2007.02.011 17449187

[B91] Seckl J. (2004). Prenatal glucocorticoids and long-term programming. Eur. J. Endocrinol. 151, U49–U62. 10.1530/eje.0.151u049 15554887

[B92] Seery M. D. Holman E. A. Silver R. C. (2010). Whatever does not kill us: cumulative lifetime adversity, vulnerability, and resilience. J. Pers. Soc. Psychol. 99, 1025–1041. 10.1037/a0021344 20939649

[B93] Seery M. D. Leo R. J. Lupien S. P. Kondrak C. L. Almonte J. L. (2013). An upside to adversity? Moderate cumulative lifetime adversity is associated with resilient responses in the face of controlled stressors. Psychol. Sci. 24, 1181–1189. 10.1177/0956797612469210 23673992

[B94] Sehlmeyer C. Schoning S. Zwitserlood P. Pfleiderer B. Kircher T. Arolt V.. (2009). Human fear conditioning and extinction in neuroimaging: a systematic review. PLoS ONE 4:e5865. 10.1371/journal.pone.0005865 19517024 PMC2692002

[B95] Seidenbecher T. Laxmi T. R. Stork O. Pape H. C. (2003). Amygdalar and hippocampal theta rhythm synchronization during fear memory retrieval. Science 301, 846–850. 10.1126/science.1085818 12907806

[B96] Shechner T. Hong M. Britton J. C. Pine D. S. Fox N. A. (2014). Fear conditioning and extinction across development: evidence from human studies and animal models. Biol. Psychol. 100, 1–12. 10.1016/j.biopsycho.2014.04.001 24746848 PMC4629237

[B97] Soztutar E. Colak E. Ulupinar E. (2016). Gender- and anxiety level-dependent effects of perinatal stress exposure on medial prefrontal cortex. Exp. Neurol. 275, 274–284. 10.1016/j.expneurol.2015.06.005 26057948

[B98] Spear L. P. (2000). The adolescent brain and age-related behavioral manifestations. Neurosci. Biobehav. Rev. 24, 417–463. 10.1016/S0149-7634(00)00014-2 10817843

[B99] Taylor S. E. (2010). Mechanisms linking early life stress to adult health outcomes. Proc. Natl. Acad. Sci. U.S.A. 107, 8507–8512. 10.1073/pnas.1003890107 20442329 PMC2889360

[B100] Tovote P. Fadok J. P. Lüthi A. (2015). Neuronal circuits for fear and anxiety. Nat. Rev. Neurosci. 16, 317–331. 10.1038/nrn3945 25991441

[B101] Treit D. Fundytus M. (1988). Thigmotaxis as a test for anxiolytic activity in rats. Pharmacol. Biochem. Behav. 31, 959–962. 10.1016/0091-3057(88)90413-3 3252289

[B102] Vallée M. Mayo W. Dellu F. Le Moal M. Simon H. Maccari S. (1997). Prenatal stress induces high anxiety and postnatal handling induces low anxiety in adult offspring: correlation with stress-induced corticosterone secretion. J. Neurosci. 17, 2626–2636. 9065522 10.1523/JNEUROSCI.17-07-02626.1997 PMC6573515

[B103] Waldherr M. Neumann I. D. (2007). Centrally released oxytocin mediates mating-induced anxiolysis in male rats. Proc. Natl. Acad. Sci. U.S.A. 104, 16681–16684. 10.1073/pnas.0705860104 17925443 PMC2034242

[B104] Weinstock M. (2008). The long-term behavioural consequences of prenatal stress. Neurosci. Biobehav. Rev. 32, 1073–1086. 10.1016/j.neubiorev.2008.03.002 18423592

[B105] Zelinski E. L. Hong N. S. Tyndall A. V. Halsall B. McDonald R. J. (2010). Prefrontal cortical contributions during discriminative fear conditioning, extinction, and spontaneous recovery in rats. Exp. Brain Res. 203, 285–297. 10.1007/s00221-010-2228-0 20449729

[B106] Zhang T. Y. Chretien P. Meaney M. J. Gratton A. (2005). Influence of naturally occurring variations in maternal care on prepulse inhibition of acoustic startle and the medial prefrontal cortical dopamine response to stress in adult rats. J. Neurosci. 25, 1493–1502. 10.1523/JNEUROSCI.3293-04.2005 15703403 PMC6725982

[B107] Zoicas I. Neumann I. D. (2016). Maternal separation facilitates extinction of social fear in adult male mice. Behav. Brain Res. 297, 323–328. 10.1016/j.bbr.2015.10.034 26497106

