# Supplementary figures and images for: Impact of Life History on Fear Memory and Extinction
Source: Front Behav Neurosci. 2016 Oct 4;10:185. doi: 10.3389/fnbeh.2016.00185 (PMC5047906; doi:10.3389/fnbeh.2016.00185)

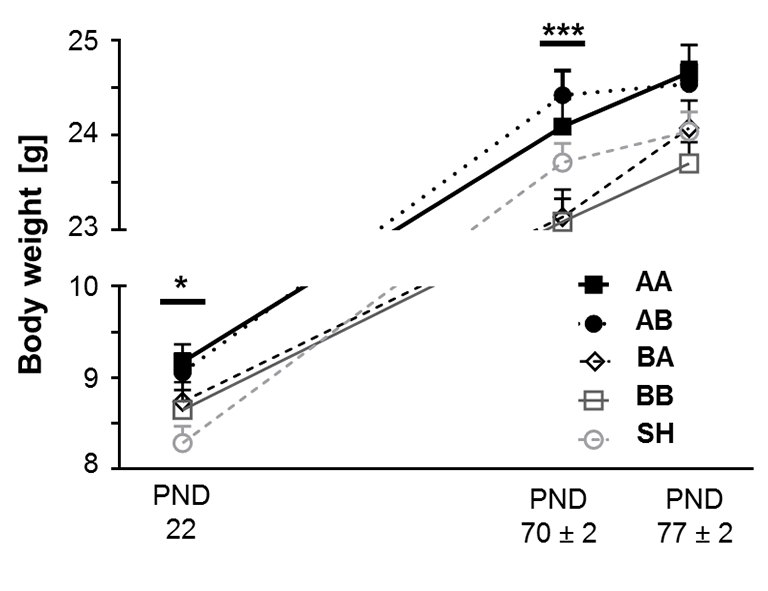

Supplement: Supplementary Figure S1 — Influence of life history on body weights. Body weights were assessed at weaning (PND 22) after simulation of either dangerous or safe environment, at the end of the early phase (PND 70 ± 2) after 5 loser or mating experiences, and at the end of the late phase (PND 77 ± 2) after escapable defeat or cohabitation with a female. Data are presented as means ±SEM. Statistics: ANOVA; post-hoc testing: ANOVA with repeated measures. *p < 0.05; ***p < 0.001. Animals per group: AA = 28, AB = 27, BA = 28, BB = 27, SH = 28. [file Image1.TIF]

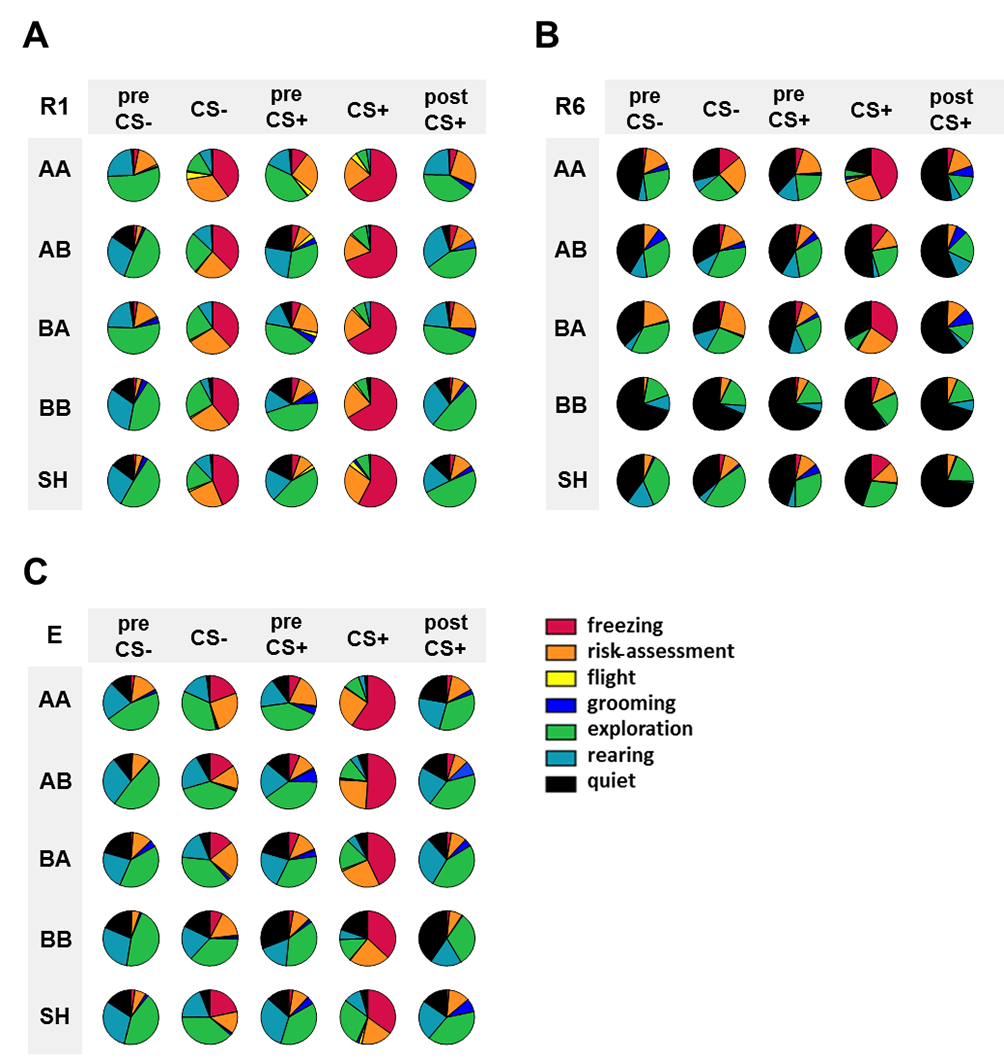

Supplement: Supplementary Figure S2 — Influence of life history on behavioral expressions after conditioned fear. (A) Fear retrieval (R1): Proportional values of displayed behaviors show a general increase of anxiety-related behaviors (freezing, risk-assessment and flight) upon CS presentations in all groups during R1 session. Non-adverse, active behaviors, such as grooming, exploration and rearing, were mostly observed prior, between and post stimulus presentations. Animals of AA and BA life history showed proportionally elevated levels of risk-assessment prior to the first stimulus presentation. (B) Fear extinction: Defensive responses became less during final session of extinction learning (R6). AA and BA mice still displayed elevated defensive behaviors during stimuli presentations. BB animals were mostly quiet. (C) Extinction recall (E): Defensive responses were mainly observed in response to stimuli presentations, whereas AA and AB mice showed a strong augmentation during CS+ presentation in comparison to other groups. Color code of different behaviors is illustrated; animals per group: n = 15. [file Image2.TIF]

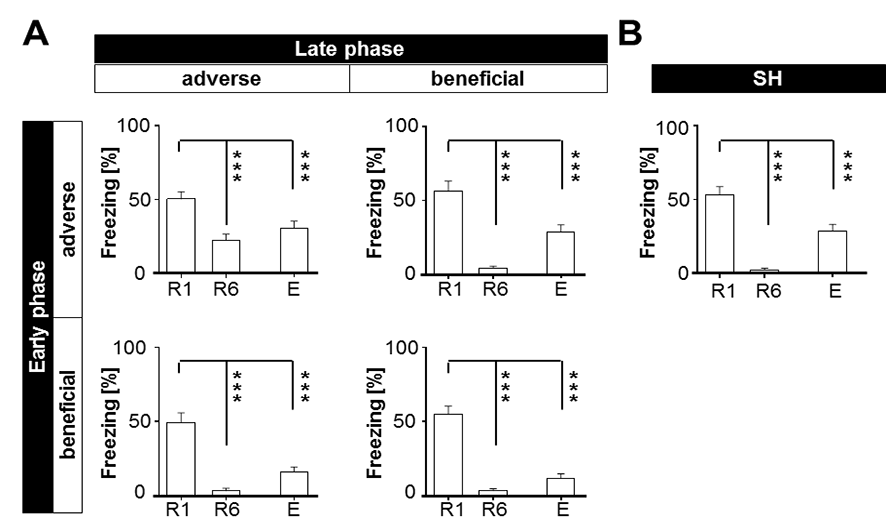

Supplement: Supplementary Figure S3 — Expression of freezing (% of time) in response to first CS− presentation during retrieval (R1), extinction (R6), and extinction recall (E) dependent on different life histories. (A) Animals of all life history groups and (B) sham-handled mice significantly reduced their freezing response to the neutral stimulus (R1 vs. R6 and E). Furthermore, comparison to freezing levels in response to the first CS+ revealed, that all groups were able to distinguish between the stimuli. Therefore, no generalization effect could be detected. ***p < 0.001; animals per group: n = 15. [file Image3.tif]
