# Supplementary material for: Impact of Life History on Fear Memory and Extinction
Source: Front Behav Neurosci. 2016 Oct 4;10:185. doi: 10.3389/fnbeh.2016.00185 (PMC5047906; doi:10.3389/fnbeh.2016.00185)
Supplement: Supplementary Table S1 — Influence of life history on discrete behavioral expressions after fear conditioning (% of time). Life histories: AA, early and late adversity; AB, early adversity and late benefit; BA, early benefit and late adversity; BB, early and late benefit; SH, sham-handled. Fear memory states: R1, retrieval session 1; R6, retrieval session 6 (end of extinction learning); E, extinction recall. Discrete behavioral expressions (freezing, risk-assessment, flight, grooming, exploration, rearing, quiet; % of time) was scored before, during and after CS− and CS+ presentation, respectively. Animals per group: n = 15. [file Table1.PDF]

R1	preCS-					CS-					preCS+					CS+					postCS+				
	AA	AB	BA	BB	SH	AA	AB	BA	BB	SH	AA	AB	BA	BB	SH	AA	AB	BA	BB	SH	AA	AB	BA	BB	SH
freezing	3.1	1.7	2.3	2.0	1.5	39.9	37.5	37.7	39.0	43.8	10.0	4.9	5.7	4.9	5.3	65.4	69.3	66.6	66.4	57.8	4.7	4.9	2.8	1.7	3.3
risk-assess.	14.9	3.5	15.0	3.6	4.3	32.5	23.3	28.2	26.8	24.3	25.1	8.2	21.9	10.9	9.2	21.1	16.8	20.7	22.1	27.5	25.6	12.3	22.3	7.8	12.0
flight	1.1	0.5	0.9	0.4	0.5	4.7	0.5	0.9	1.0	1.3	3.3	3.2	2.7	1.2	2.2	4.0	1.0	1.4	1.5	3.3	0.3	0.0	0.0	0.0	0.0
grooming	0.7	3.3	3.2	1.4	2.8	0.7	0.3	0.2	0.0	0.0	0.6	3.3	4.5	7.1	0.3	0.1	0.0	0.1	0.0	1.8	3.6	5.4	5.2	3.1	3.2
exploration	54.2	43.9	52.4	48.4	49.1	13.6	25.5	23.1	25.5	18.5	41.8	32.8	42.4	45.7	45.0	6.7	10.3	7.4	7.5	8.8	39.7	42.3	45.4	48.5	49.1
rearing	24.1	31.7	21.5	28.8	27.1	7.8	12.3	9.0	5.3	10.8	16.1	25.3	15.0	14.6	20.4	2.6	1.8	3.7	0.8	1.0	23.2	30.0	20.6	29.1	19.3
quiet	1.5	15.3	2.4	15.4	14.7	0.8	0.8	0.4	2.5	1.5	1.5	22.3	7.0	15.6	17.5	0.0	1.0	0.0	1.8	0.0	0.8	5.0	2.4	9.9	13.1

R6	preCS-					CS-					preCS+					CS+					postCS+				
	AA	AB	BA	BB	SH	AA	AB	BA	BB	SH	AA	AB	BA	BB	SH	AA	AB	BA	BB	SH	AA	AB	BA	BB	SH
freezing	2.0	0.4	0.4	0.1	0.5	13.8	3.5	3.4	1.1	3.4	4.9	3.3	4.6	1.9	3.6	43.4	10.4	34.7	4.9	13.2	4.3	0.3	0.5	0.1	0.0
risk-assess.	15.8	9.5	20.1	2.6	5.7	23.5	15.3	26.7	5.8	10.3	18.9	9.2	11.1	6.4	10.7	26.5	11.7	23.4	12.9	13.1	15.3	5.6	12.4	6.1	5.4
flight	0.1	0.0	0.0	0.0	0.0	0.2	0.5	0.3	0.0	0.0	0.8	0.5	0.0	0.1	0.3	1.9	0.3	0.4	0.6	0.0	0.0	0.0	0.0	0.0	0.0
grooming	3.7	7.1	1.0	0.1	1.2	0.7	3.3	0.7	0.5	1.5	1.0	3.8	2.4	0.0	5.2	1.7	0.0	0.6	0.0	0.6	6.9	6.7	9.6	0.1	0.8
exploration	26.2	30.9	36.0	16.7	36.1	25.5	34.5	26.5	18.7	44.1	22.4	30.6	25.1	16.1	30.2	4.6	22.8	8.1	21.1	28.2	14.8	19.5	13.0	16.3	19.2
rearing	5.5	10.7	5.0	9.7	16.6	7.2	9.7	12.4	5.3	4.8	13.8	11.1	10.7	5.3	4.9	0.9	3.5	1.1	1.4	0.8	5.9	11.5	4.1	7.0	1.3
quiet	46.7	41.4	37.5	70.8	39.9	29.1	33.2	29.3	68.7	36.0	38.2	41.5	46.1	70.2	45.2	21.2	51.2	31.8	59.1	44.1	52.8	56.4	60.4	70.2	73.3

E	preCS-					CS-					preCS+					CS+					postCS+				
	AA	AB	BA	BB	SH	AA	AB	BA	BB	SH	AA	AB	BA	BB	SH	AA	AB	BA	BB	SH	AA	AB	BA	BB	SH
freezing	2.0	0.8	1.2	0.3	2.3	19.7	15.5	14.1	7.4	21.7	7.3	6.3	6.1	2.6	2.4	58.0	51.1	42.9	37.1	35.1	3.2	4.5	2.6	1.5	1.3
risk-assess.	15.1	10.5	11.4	4.8	6.6	26.7	13.8	21.7	15.8	13.3	20.6	10.6	12.4	10.0	9.5	25.7	24.8	25.2	23.6	18.4	12.7	8.2	9.2	7.5	12.6
flight	0.1	0.1	0.1	0.0	0.0	0.7	0.9	1.0	0.7	0.4	0.4	0.8	0.7	0.5	0.4	0.4	0.7	1.1	0.3	1.8	0.0	0.0	0.0	0.0	0.0
grooming	1.9	0.5	3.9	1.0	1.7	1.0	0.6	1.7	1.5	1.2	4.8	7.7	3.7	1.7	4.3	0.1	0.5	0.0	0.1	1.3	2.8	8.2	4.5	0.5	7.8
exploration	45.8	48.1	39.8	46.4	43.3	34.3	39.6	38.0	36.5	38.5	40.0	39.5	34.2	36.8	38.1	10.2	12.5	18.3	13.3	28.7	35.5	39.2	42.2	32.0	39.2
rearing	22.6	29.6	23.0	28.9	30.4	15.6	21.5	17.4	20.4	19.0	16.4	21.3	22.5	17.4	32.0	3.9	4.6	5.1	5.9	10.3	25.2	23.0	30.2	18.0	23.8
quiet	12.4	10.4	20.5	18.6	15.7	2.0	8.1	6.1	17.8	5.9	10.4	13.8	20.3	31.0	13.3	1.8	5.8	7.3	19.7	4.4	20.6	16.8	11.4	40.5	15.2

Values in % total time
